# Loss of TLR3 aggravates CHIKV replication and pathology due to an altered virus-specific neutralizing antibody response

**DOI:** 10.15252/emmm.201404459

**Published:** 2014-12-01

**Authors:** Zhisheng Her, Terk-Shin Teng, Jeslin JL Tan, Teck-Hui Teo, Yiu-Wing Kam, Fok-Moon Lum, Wendy WL Lee, Christelle Gabriel, Rossella Melchiotti, Anand K Andiappan, Valeria Lulla, Aleksei Lulla, Mar K Win, Angela Chow, Subhra K Biswas, Yee-Sin Leo, Marc Lecuit, Andres Merits, Laurent Rénia, Lisa FP Ng

**Affiliations:** 1 Singapore Immunology Network (SIgN), Agency for Science, Technology and Research (A*STAR), BiopolisSingapore, Singapore; 2 Department of Biochemistry, Yong Loo Lin School of Medicine, National University of SingaporeSingapore, Singapore; 3 NUS Graduate School for Integrative Sciences and Engineering, National University of SingaporeSingapore, Singapore; 4 Doctoral School in Translational and Molecular Medicine (DIMET), University of Milano-BicoccaMilan, Italy; 5 Institute of Technology, University of TartuTartu, Estonia; 6 Institute of Infectious Disease and Epidemiology (IIDE), Tan Tock Seng HospitalSingapore, Singapore; 7 Institut Pasteur, Biology of Infection UnitParis, France; 8 Inserm U1117Paris, France; 9 Paris Descartes University, Sorbonne Paris Cité, Necker-Enfants Malades University Hospital, Institut ImagineParis, France; 10 Institute of Infection and Global Health, University of LiverpoolLiverpool, UK

**Keywords:** Chikungunya virus, innate immunity, joint inflammation, neutralizing antibodies, TLR3

## Abstract

RNA-sensing toll-like receptors (TLRs) mediate innate immunity and regulate anti-viral response. We show here that TLR3 regulates host immunity and the loss of TLR3 aggravates pathology in Chikungunya virus (CHIKV) infection. Susceptibility to CHIKV infection is markedly increased in human and mouse fibroblasts with defective TLR3 signaling. Up to 100-fold increase in CHIKV load was observed in *Tlr3*^*−/−*^ mice, alongside increased virus dissemination and pro-inflammatory myeloid cells infiltration. Infection in bone marrow chimeric mice showed that TLR3-expressing hematopoietic cells are required for effective CHIKV clearance. CHIKV-specific antibodies from *Tlr3*^*−/−*^ mice exhibited significantly lower *in vitro* neutralization capacity, due to altered virus-neutralizing epitope specificity. Finally, SNP genotyping analysis of CHIKF patients on *TLR3* identified SNP rs6552950 to be associated with disease severity and CHIKV-specific neutralizing antibody response. These results demonstrate a key role for TLR3-mediated antibody response to CHIKV infection, virus replication and pathology, providing a basis for future development of immunotherapeutics in vaccine development.

## Introduction

Innate immunity against RNA viruses involves pattern recognition receptors (PRRs) that recognize structurally conserved molecules from diverse pathogens known as pathogen-associated molecular patterns (PAMPs) (Arpaia & Barton, 2011). PRRs include TLRs (particularly TLR3, TLR7 and TLR8) and members of the cytosolic retinoic acid-inducible gene I (RIG-I)-like receptors such as melanoma differentiation-associated protein 5 (MDA5) and RIG-I that detect RNA viruses through their genomic RNA or the double-stranded RNA (dsRNA) viral intermediate generated during replication (Yoneyama *et al*, 2004, 2005; Gitlin *et al*, 2006). Activation of these PRRs induces downstream anti-viral type I IFN response, which can also occur independently of viral RNA transcription and replication (Nikonov *et al*, 2013).

The involvement of TLRs in counteracting RNA virus infection is widely documented (Arpaia & Barton, 2011; Neighbours *et al*, 2012; Zhang *et al*, 2013). TLR3 recognizes dsRNA and can influence disease outcomes depending on the type of virus and infection model. TLR3-mediated innate and inflammatory responses were demonstrated to be protective against HIV, CMV and Dengue virus infections, while TLR3 stimulation results in detrimental disease outcomes in Influenza A virus and Punta Toro virus infections (Tabeta *et al*, 2004; Goffic *et al*, 2006; Gowen *et al*, 2006; Suh *et al*, 2007; Nasirudeen *et al*, 2011). TLR3-dependent response has been shown to be both protective by restricting virus replication in neurons (Daffis *et al*, 2008) and also detrimental in West Nile virus infection by perturbing TNFR1 signaling to promote virus entry into the brains of mice resulting in lethal encephalitis (Wang *et al*, 2004). Clinically, patients with impaired TLR3-mediated responses show an increased susceptibility to HSV-1 encephalitis (Zhang *et al*, 2007; Pérez de Diego *et al*, 2010; Sancho-Shimizu *et al*, 2011). Repeated reactivation of HSV-2 that led to the development of Mollaret meningitis has also been reported in an individual with TLR3 deficiency (Willmann *et al*, 2010).

The significance of TLR-mediated signaling and how TLR molecules influence clinically important re-emerging viruses such as CHIKV remains confounding. CHIKV is an ‘Old World’ alphavirus with a positive sense RNA genome belonging to the *Togaviridae* family (Deller & Russell, 1968). CHIKV is the causative agent for CHIKF, and over the last decade, it has caused simultaneous outbreaks of unprecedented scale in the Indian Ocean Islands (Josseran *et al*, 2006), India (Kaur *et al*, 2008) and subsequently in South East Asia (Laras *et al*, 2005; AbuBakar *et al*, 2007; Leo *et al*, 2009) and Europe (Queyriaux *et al*, 2008). Serious CHIKF outbreaks have also occurred in Cambodia (Centers for Disease Control & Prevention, 2012; Duong *et al*, 2012), Laos (Soulaphy *et al*, 2013) and Sierra Leone (Ansumana *et al*, 2013). Since 2013, it has finally reached the Americas and triggered ongoing outbreaks in the French West Indies (Enserink, 2014; Leparc-Goffart *et al*, 2014). The clinical presentation of the disease is characterized by flu-like symptoms such as fever, rash and muscle aches which subside in 7–10 days (Kam *et al*, 2009). The hallmark of CHIKV infection is the incapacitating arthralgia that routinely persists for weeks or months after resolution of the acute symptoms and has high costs in terms of both quality of life and healthcare provision/economic loss (Borgherini *et al*, 2008).

The involvement of TLRs in CHIKV replication was previously investigated in murine models (Schilte *et al*, 2010, 2012), but their role in CHIKV pathology and dissemination was not well established. We show here that TLR3 signaling plays a critical role in the control of CHIKV infection, replication, dissemination and pathology. Complementing an earlier report where CHIKV-infected *Trif*^*−/−*^ (Toll/IL-1 resistance domain-containing adaptor inducing IFNβ; an adaptor protein essential for TLR3-mediated signaling) mice showed more pronounced viremia and joint inflammation compared to WT mice (Rudd *et al*, 2012), this study further demonstrated that infection of cultured primary human *TRIF*^*−/−*^ and mouse *Tlr3*^*−/−*^ fibroblasts resulted in a significant enhancement of virus replication. Notably, infected *Tlr3*^*−/−*^ mice developed higher viremia and more pronounced joint inflammation, associated with a massive infiltration of myeloid cells such as neutrophils and macrophages when compared to WT mice. Furthermore, monitoring of virus infection using a firefly luciferase (FLuc)-tagged recombinant CHIKV (FLuc-CHIKV) revealed increased CHIKV dissemination in *Tlr3*^*−/−*^ mice. By infecting bone marrow chimeric mice, we showed that TLR3-expressing hematopoietic cells were required for effective CHIKV clearance, but did not directly regulate CHIKV-induced joint inflammation. Mechanistic investigations further demonstrated that TLR3 was required by hematopoietic cells to direct CHIKV-specific antibody response toward important neutralizing linear B-cell epitopes in the E2 glycoprotein. In the absence of TLR3, high levels of CHIKV-specific IgG were still generated, but with substantially diminished neutralizing capacity. The clinical relevance of TLR3 was further investigated in CHIKV-infected patients, where the level of *TLR3* transcripts was increased in PBMCs of CHIKV-infected patients. Interestingly, SNP genotyping analysis further identified *TLR3* SNPs rs3775292 and rs6552950, whose functional effects remain unknown, to be associated with prevalence of CHIKV phenotypes, and in the case of SNP rs6552950, also with disease severity, CHIKV-specific IgG response and antibody neutralizing capacity. Taken together, these results substantiate a role for TLR3 in the control of CHIKV replication, immunity and pathology.

## Results

### TRIF deficiency increases CHIKV replication

**Figure 1:**
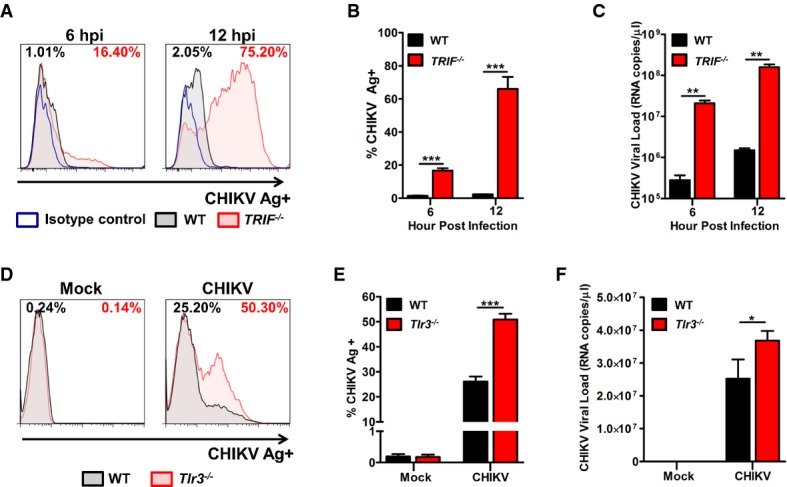
Enhanced CHIKV replication in *TRIF*^*−/−*^ and *Tlr3*^*−/−*^ primary fibroblasts from human and mice, respectively A–C CHIKV replication in *TRIF*^*−/−*^ primary human fibroblasts was determined by CHIKV Ag detection using flow cytometry (A and B) and viral load quantification using qRT-PCR (C) after 6 and 12 hpi (MOI 10). Infection was performed in triplicate, and data are representative of two independent experiments and presented as mean ± SD (two-tailed unpaired *t-*test, ***P = 0.0005 6 hpi CHIKV Ag, ***P =* *0.0009 12 hpi CHIKV Ag, **P =* *0.0041 6 hpi viral load, **P =* *0.0043 12 hpi viral load).D–F Primary tail fibroblasts isolated from WT and *Tlr3*^*−/−*^ mice (*n *=* *3 per group) were infected with CHIKV (MOI 10). CHIKV infectivity was determined by flow cytometry for CHIKV Ag (D and E) and by qRT-PCR for viral load quantification (F) at 12 hpi. Infection was performed in triplicate, and data are representative of two independent experiments and presented as mean ± SD (two-tailed unpaired *t-*test, ***P =* *0.0001 CHIKV Ag, *P =* *0.0368 viral load).

Activation of various TLR signaling pathways including TLR2, TLR3 and TLR4 engage the TRIF adaptor protein to induce expression of downstream anti-viral and pro-inflammatory genes (Yamamoto *et al*, 2003). To demonstrate a functional role for TRIF signaling in anti-CHIKV response, human primary fibroblasts with homozygous *TRIF* nonsense mutation (Sancho-Shimizu *et al*, 2011) were infected with CHIKV *in vitro*. CHIKV replication was remarkably higher in the *TRIF*^*−/−*^ fibroblasts with a 2-log difference when compared to healthy control (Fig1A–C). This observation is in line with previous findings where *TRIF*^*−/−*^ fibroblasts were shown to be more susceptible to infection with HSV-1 and vesicular stomatitis virus (Sancho-Shimizu *et al*, 2011) due to a defect in type I interferon induction. This suggested that TLRs could be involved in CHIKV infection. We focused our study on TLR3 because TLR3-mediated immunity and polymorphisms to viral infection in human have been demonstrated to regulate disease progression (Pérez de Diego *et al*, 2010; Reinert *et al*, 2012; Zhang *et al*, 2007b).

### TLR3 inhibits CHIKV replication

To dissect the significance of TLR3-mediated anti-CHIKV response, primary fibroblasts were isolated from both WT and *Tlr3*^*−/−*^ mice and infected with CHIKV *ex vivo*. The loss of TLR3 significantly increased susceptibility to CHIKV infection that led to marked virus replication when compared to WT fibroblasts (Fig1D–F). To gain further insights on how TLR3 signaling mediates an anti-CHIKV state, transcriptional profiles of type I IFNs and related IFN-stimulated genes (ISGs) during infection were analyzed (Supplementary Fig S1). Results revealed that the induction of type I IFNs were higher in *Tlr3*^*−/−*^ fibroblasts, suggesting that the induction of type I IFN response observed here is independent of TLR3.

### Loss of TLR3 leads to more pronounced virus dissemination and CHIKV-induced pathology

**Figure 2:**
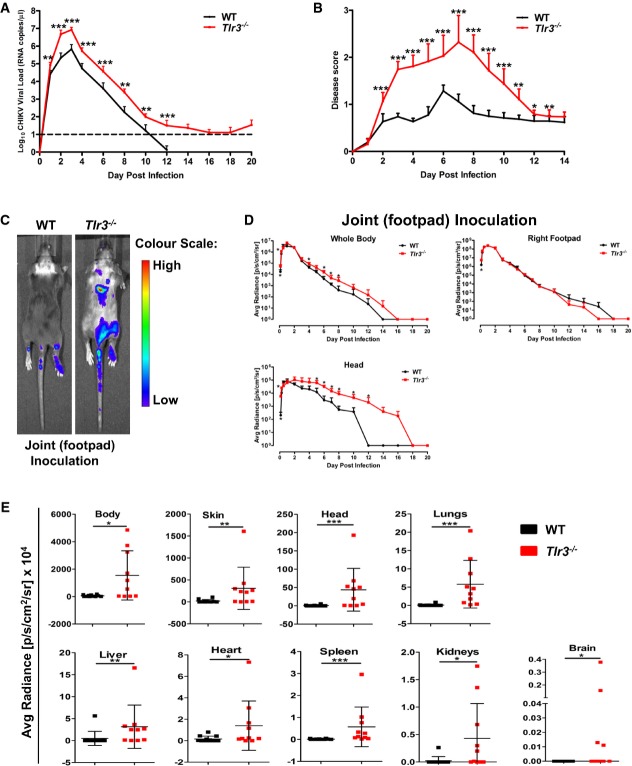
TLR3 modulates CHIKV replication, disease pathology and dissemination in mice A, B WT and *Tlr3*^*−/−*^ mice (*n *=* *5 per group) were infected with CHIKV (10^6^ PFU) by joint footpad inoculation. (A) Viremia was determined from blood collected from tails by viral load quantification. Dotted line indicates the limit of viral load detection. (B) Extent of joint inflammation was measured daily and expressed as disease score relative to day 0 (pre-infection). Data are representative of three independent experiments and presented as mean ± SD (two-tailed Mann–Whitney *U*-test, **P =* *0.0037 1 dpi viremia, ***P =* *0.0006 2 dpi viremia, ***P =* *0.0003 3–6 dpi viremia, **P =* *0.0012 8 dpi viremia, **P =* *0.0012 10 dpi viremia, ***P =* *0.0003 12 dpi viremia, ***P =* *0.0002 2–9 dpi disease score, ***P =* *0.0003 10 dpi disease score, **P =* *0.003 11 dpi disease score, *P =* *0.014 12 dpi disease score, **P =* *0.0093 13 dpi disease score).C, D TLR3 modulates CHIKV dissemination in mice. WT and *Tlr3*^*−/−*^ mice were infected with FLuc-CHIKV (10^6^ PFU) by joint footpad (*n *=* *4–5 per group) inoculation. Bioluminescence signals were measured using an *in vivo* bioluminescence imaging system. (C) Images of representative 6 dpi in WT and *Tlr3*^*−/−*^ mice. Color scale indicates the level of bioluminescence signals detected. (D) Bioluminescence signals of whole body, head region and at site of inoculation were quantified and expressed as average radiance (p/s/cm^2^/sr). The lowest limit of detection is 0 p/s/cm^2^/sr. Data are representative of two independent experiments and presented as mean ± SD (two-tailed Mann–Whitney *U*-test, *P =* *0.0159 3 hpi whole body, *P =* *0.0317 6 hpi whole body, *P =* *0.0317 4 dpi whole body, *P =* *0.0159 6 dpi whole body, *P =* *0.0159 7 dpi whole body, *P =* *0.0317 8 dpi whole body, *P =* *0.0195 3 hpi right footpad, *P =* *0.0159 3 hpi head, *P =* *0.0159 6 hpi head, *P =* *0.0317 5 dpi head, *P =* *0.0159 6 dpi head, *P =* *0.0317 7 dpi head, *P =* *0.0317 8 dpi head, *P =* *0.0317 10 dpi head, *P =* *0.0476 12 dpi head).E TLR3 modulates CHIKV tissue tropism in mice. Bioluminescence signals of various organs, body and skin of WT and *Tlr3*^*−/−*^ mice (*n = *10–12 per group) infected with FLuc-CHIKV (10^6^ PFU) by joint footpad inoculation at 3 dpi were quantified and expressed as average radiance (p/s/cm^2^/sr). The lowest limit of detection is 0 p/s/cm^2^/sr. Data are representative of two independent experiments and presented as mean ± SD (two-tailed Mann–Whitney *U*-test, *P =* *0.0206 body, **P =* *0.0034 skin, ***P =* *0.0003 head, ***P =* *0.0002 lungs, **P =* *0.0011 liver, *P =* *0.0434 heart, ***P =* *0.0003 spleen, *P =* *0.0117 kidneys, *P =* *0.0213 brain).

The importance of TLR3 in CHIKV infection was next examined *in vivo* in both WT and *Tlr3*^*−/−*^ mice via the joint footpad inoculation route (Gardner *et al*, 2010; Teng *et al*, 2012). Animals were monitored daily for survival, viremia and joint inflammation. Although the loss of TLR3 expression did not affect animal survival, significantly higher viremia was observed throughout the course of disease in *Tlr3*^*−/−*^animals (Fig2A). Strikingly, *Tlr3*^*−/−*^ mice exhibited a remarkable exacerbation of CHIKV-induced inflammation at the joint footpad (Fig2B). Transcriptional analysis on joint footpad samples harvested at the peak of viremia revealed that the induction of type I IFNs was differentially induced in *Tlr3*^*−/−*^ mice as compared to WT mice (Supplementary Fig S2).

**Figure 3:**
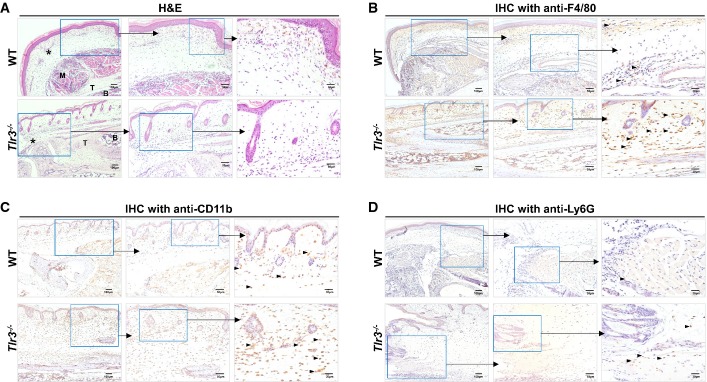
Loss of TLR3 increases myeloid cells infiltration at the peak of joint swelling A–D WT and *Tlr3*^*−/−*^ mice (*n *=* *5–6 per group) were infected with CHIKV (10^6^ PFU) by joint footpad inoculation. Histological analysis of CHIKV-inoculated joint footpad samples from WT and *Tlr3*^*−/−*^ mice by H&E staining (A) and labeling with anti-F4/80 antibody (B), anti-CD11b (C) and anti-Ly6G (D) at 6 dpi. Boxed regions are shown at higher magnification on the right. * = edema, B = bone, M = muscle, T = tendon. Black arrow heads indicate positively stained cells. Images presented are from one mouse representative of 3 mice per group from two independent experiments. Scale bars: left, 100 μm; middle, 50 μm; right, 30 μm.

To further study the role of TLR3 on *in vivo* CHIKV replication and dissemination, we used a recombinant FLuc-CHIKV to infect both WT and *Tlr3*^*−/−*^ mice and tracked the kinetics of CHIKV infection by live imaging for a duration of 20 dpi. As expected, the loss of TLR3 once again resulted in more pronounced joint inflammation and viremia (Supplementary Fig S3A–C). Bioluminescence signals indicative of virus replication distinctly reveal the differences in the route of CHIKV dissemination in WT and *Tlr3*^*−/−*^ mice during the initial phase of infection (Fig3C and Supplementary Videos S1 and S2). From the site of infection (i.e. right joint footpad), signals were next detected in the posterior regions (tail, lymph node and left footpad) before moving to the anterior regions in the lower abdomen and head (Supplementary Videos S1 and S2). Notably, CHIKV dissemination was more rapid and extensive in *Tlr3*^*−/−*^ mice than in WT mice (Fig2C and D and Supplementary Videos S1 and S2). High level of virus replication was detected from the whole body by the bioluminescence signals (Fig2D) and also from the blood by viral RNA quantification (Supplementary Fig S3C). The *Tlr3*^*−/−*^ mice exhibited delayed virus clearance throughout the disease. On the contrary, bioluminescence signals were mainly localized at the site of infection in WT mice (Fig2C and Supplementary Video S1). Notably, bioluminescence signals peaked at 2–3 dpi in the head region of *Tlr3*^*−/−*^ mice and remained detectable until 16 dpi (Fig2D).

To determine whether the pattern of CHIKV dissemination was specific to the loss of TLR3 and not due to the site of virus inoculation, mice were subjected to s.c. inoculation of CHIKV at the dermis region of the ear prior to live imaging analysis (Supplementary Fig S4 and Supplementary Videos S3 and S4). Similar patterns of CHIKV dissemination were observed. Although the level of bioluminescence signals measured by the ear inoculation route was lower, signals detected from the whole body, head region and inoculated ear in *Tlr3*^*−/−*^ mice still remained significantly higher than that of the WT mice (Supplementary Fig S4B and Supplementary Videos S3 and S4) and correlated with viremia (Supplementary Fig S3D).

To further evaluate whether a loss of TLR3 expression affects virus tropism in tissues and deeper organs, mice inoculated via the joint footpad route were sacrificed at 3 dpi and dissected in order to quantify bioluminescence signals from the various organs, skeletal body and skin (Fig2E). Higher bioluminescence signals were detected from the skeletal body, skin, head, lungs, liver, heart, spleen, kidneys and brain of *Tlr3*^*−/−*^ mice (Fig2E), confirming the role of TLR3 in controlling CHIKV infection and virus dissemination. Interestingly, bioluminescence signals were also detected in the brain in 40% of *Tlr3*^*−/−*^ mice (Fig2E), and positive bioluminescence signals were still detectable in the body, skin and head of *Tlr3*^*−/−*^ mice at 6 dpi (Supplementary Fig S5).

### Severe joint inflammation in *Tlr3*^*−/−*^ mice is associated with a massive infiltration of myeloid cells

**Figure 4:**
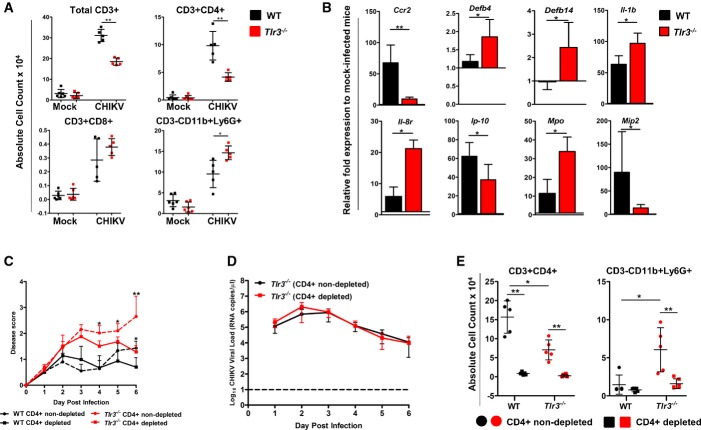
Loss of TLR3 modulates neutrophils recruitment, but not CD4^+^ T-cell-mediated joint inflammation A WT and *Tlr3*^*−/−*^ mice were infected with CHIKV (10^6^ PFU) by joint footpad (*n *=* *5–6 per group) inoculation. At 6 dpi, cells from the CHIKV-inoculated footpad were harvested and labeled for CD45, CD3, CD4, CD8, CD11b and Ly6G. The absolute cell counts of each immune cell subset were calculated based on the total number of live cells determined before labeling. Data are representative of one of two independent experiments and presented as mean ± SD (two-tailed Mann–Whitney *U*-test, **P =* *0.0079 total CD3^+^, **P =* *0.0079 CD3^+^CD4^+^, *P =* *0.0159 CD3^−^CD11b^+^Ly6G^+^).B Increased recruitment of neutrophils in *Tlr3*^*−/−*^ mice on 6 dpi was associated with induction of neutrophil-associated molecules/chemokines expression. WT and *Tlr3*^*−/−*^mice (*n *=* *5–6 per group) were infected with CHIKV (10^6^ PFU) by joint footpad inoculation. The level of gene expression was expressed as fold change compared to mock-infected mice footpad (*n *=* *5 per group) after normalization to *Gapdh*. Data are representative of two independent experiments and presented as mean ± SD (two-tailed Mann–Whitney *U*-test, **P =* *0.0079 *Ccr2*, *P =* *0.317 *Defb4*, *P =* *0.0159 *Defb14*, *P =* *0.0317 *Il-1b*, *P =* *0.0159 *Il-8r*, *P =* *0.0317 *Ip-10*, *P =* *0.0159 *Mpo*, *P =* *0.0317 *Mip2*).C, D Depletion of CD4^+^ T cells in *Tlr3*^*−/−*^ mice reduced severity of joint swelling. *Tlr3*^*−/−*^ mice were injected i.p. with rat anti-mouse CD4 antibody to deplete CD4^+^ T cells on −2 and −1 dpi before CHIKV infection (10^6^ PFU) by joint footpad (*n *=* *5–6 per group) inoculation. (C) Extent of joint inflammation was measured daily and expressed as disease score relative to day 0 (pre-infection), and (D) viremia was determined by viral load quantification. Dotted line indicates the limit of viral load detection. Data are representative of two independent experiments and presented as mean ± SD (two-tailed Mann–Whitney *U*-test, *P =* *0.0159 6 dpi CD4^+^ T-cell-depleted and T-cell-non-depleted WT mice, *P =* *0.0173 4 dpi CD4^+^ T-cell-depleted and T-cell-non-depleted *Tlr3*^*−/−*^ mice, *P =* *0.0281 5 dpi CD4^+^ T-cell-depleted and T-cell-non-depleted *Tlr3*^*−/−*^ mice, **P =* *0.0087 6 dpi CD4^+^ T-cell-depleted and T-cell-non-depleted *Tlr3*^*−/−*^ mice).E Depletion of CD4^+^ T cells reduced recruitment of neutrophils in *Tlr3*^*−/−*^ mice. At 6 dpi, cells from CHIKV-inoculated footpad were harvested and labeled for CD45, CD3, CD4, CD11b and Ly6G. Absolute cell counts of each immune cell subset were calculated according to the total number of live cells determined before labeling. Data are representative of two independent experiments and presented as mean ± SD (two-tailed Mann–Whitney *U*-test, *P =* *0.0159 CD3^+^CD4^+^ of CD4^+^ T-cell-non-depleted WT and *Tlr3*^*−/−*^ mice, **P =* *0.0079 CD3^+^CD4^+^ of CD4^+^ T-cell-depleted and T-cell-non-depleted WT mice, **P =* *0.0043 CD3^+^CD4^+^ of CD4^+^ T-cell-depleted and T-cell-non-depleted *Tlr3*^*−/−*^ mice, *P =* *0.0317 CD3^−^CD11b^+^Ly6G^+^ of CD4^+^ T-cell-non-depleted WT and *Tlr3*^*−/−*^ mice, **P =* *0.0043 CD3^−^CD11b^+^Ly6G^+^ of CD4^+^ T-cell-depleted and T-cell-non-depleted *Tlr3*^*−/−*^ mice).

In an effort to assess tissue damage in the joints of CHIKV-infected mice, histological assessments revealed an intensified disease score in *Tlr3*^*−/−*^ mice that correlated with more pronounced subcutaneous edema and infiltration of CD11b^+^ myeloid cells at 3 dpi (Supplementary Fig S6) and during the peak of disease severity at 6 dpi (Fig3). The infiltrated myeloid cells consisted primarily of F4/80^+^ macrophages that were localized in the edema region, while the Ly6G^+^ neutrophils were localized distinctly in the skeletal muscles. In addition, cellular infiltrates in the joint of virus-infected mice were further analyzed during the peak of inflammation at 6 dpi by flow cytometry. Consistent with histological analysis (Fig3), the loss of TLR3 resulted in a significant increase in infiltrating CD11b^+^ myeloid cells and, in particular, CD11b^+^Ly6G^+^ neutrophils in *Tlr3*^*−/−*^ mice (Fig4A and Supplementary Fig S7). This observation was further complemented by transcriptional analysis of the CHIKV-infected joint footpad that revealed a significant induction of neutrophil-associated activation molecules and chemokines such as defensins *Defb4*,*Defb14*, myeloperoxidase (*Mpo*), *Il-1b* and *Il-8r* in *Tlr3*^*−/−*^ mice at 6 dpi (Fig4B).

Although both CD4^+^ and CD8^+^ T cells were previously demonstrated to infiltrate the joint footpad, only CD4^+^ T cells were responsible for the pathology (Teo *et al*, 2013). Therefore, it would be compelling to assess the number of CD4^+^ T cells in *Tlr3*^*−/−*^ mice. Interestingly, the number of infiltrating CD4^+^ T cells in the joint footpad was significantly reduced by half (Fig4A). Joint inflammation was significantly reduced both in CHIKV-infected WT and *Tlr3*^*−/−*^ mice depleted of CD4^+^ T cells when compared to controls (Fig4C), indicating that joint pathology is mediated by CD4^+^ T cells in both groups. However, depleting CD4^+^ T cells did not modify the viremia in both WT and *Tlr3*^*−/−*^ mice (Figure4D), demonstrating that virus replication is independent of CD4^+^ T cells. Furthermore, CD4^+^ T-cell depletion resulted in a significant reduction in Ly6G^+^ neutrophils infiltration at 6 dpi (Fig4E), supporting earlier observations (Figure3) that neutrophils are a vital mediator of footpad joint inflammation in *Tlr3*^*−/−*^ mice.

### TLR3-expressing hematopoietic cells are required for effective CHIKV clearance

**Figure 5:**
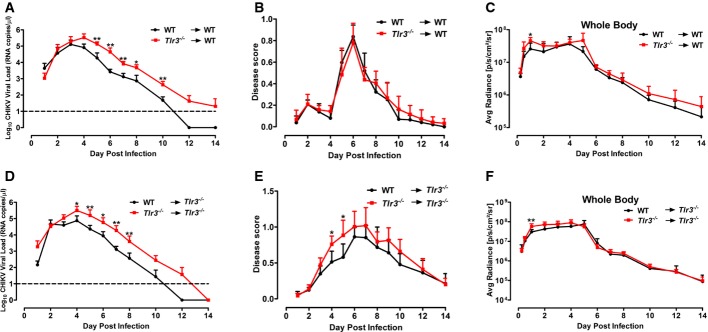
*Tlr3*^*−/−*^→WT and *Tlr3*^*−/−*^→*Tlr3*^*−/−*^ bone marrow chimeras were impaired in CHIKV clearance A, D WT and *Tlr3*^*−/−*^ mice (*n *=* *4–7 per group) were lethally irradiated and reconstituted with bone marrow cells from WT or *Tlr3*^*−/−*^mice. Chimeric mice were infected with CHIKV (10^6^ PFU) by joint footpad inoculation after 6 wks. Viremia was determined by viral load quantification. Dotted line indicates the limit of viral load detection. Data are representative of two independent experiments and presented as mean ± SD (two-tailed Mann–Whitney *U*-test, **P =* *0.0095 5–7 dpi viremia WT recipient chimera, *P =* *0.0139 8 dpi viremia WT recipient chimera, **P =* *0.0095 10 dpi viremia WT recipient chimera, *P =* *0.0350 4 dpi viremia *Tlr3*^*−/−*^ recipient chimera, **P =* *0.0082 5 dpi viremia *Tlr3*^*−/−*^ recipient chimera, *P =* *0.0221 6 dpi viremia *Tlr3*^*−/−*^ recipient chimera, **P =* *0.0012 7 dpi viremia *Tlr3*^*−/−*^ recipient chimera, **P =* *0.0082 8 dpi viremia *Tlr3*^*−/−*^ recipient chimera).B, E Extent of joint inflammation was measured daily and expressed as disease score relative to day 0 (pre-infection). Data are representative of two independent experiments and presented as mean ± SD (two-tailed Mann–Whitney *U*-test, *P =* *0.0117 4 dpi disease score *Tlr3*^*−/−*^ recipient chimera, *P =* *0.0146 5 dpi disease score *Tlr3*^*−/−*^ recipient chimera).C, F CHIKV dissemination is not modulated in WT and *Tlr3*^*−/−*^ chimeras. Bioluminescence signals of whole body was quantified and expressed as average radiance (p/s/cm^2^/sr). The lowest limit of detection is 0 p/s/cm^2^/sr. Data are representative of one of two independent experiments and presented as mean ± SD (two-tailed Mann–Whitney *U*-test, *P =* *0.0381 1 dpi whole-body WT recipient chimera, **P =* *0.0082 1 dpi whole-body *Tlr3*^*−/−*^ recipient chimera).

It is well established that hematopoietic and non-hematopoietic cells are engaged in the control of CHIKV infection by the innate immune system (Her *et al*, 2010; Schilte *et al*, 2010). To next assess whether effective CHIKV clearance could be mediated directly by TLR3-expressing hematopoietic cells, bone marrow chimeric mice were generated by lethal irradiation of WT mice, followed by adoptive transfer of either WT (referred to as WT→WT) or *Tlr3*^*−/−*^ (referred to as *Tlr3*^*−/−*^→WT) bone marrow cells. Upon successful bone marrow engraftment after 6 weeks, chimeric mice were infected with recombinant FLuc-CHIKV by footpad inoculation and monitored for joint inflammation, viremia and CHIKV dissemination. Strikingly, *Tlr3*^*−/−*^→WT chimeras suffered significantly higher viremia from 5 dpi onward and endured delayed virus clearance till 14 dpi (Fig5A), but joint inflammation and bioluminescence signals detected in body and CHIKV-infected footpad were not significantly different when compared to WT→WT chimeras (Fig5B and C). The similar degree of joint inflammation observed between these two groups further substantiate that the differences observed in joint inflammation between WT and *Tlr3*^*−/−*^ mice were not mediated by TLR3 deficiency in hematopoietic cells. Nonetheless, these results indicate that TLR3-expressing hematopoietic cells are the main subsets required for effective CHIKV clearance. This notion was further supported by the converse bone marrow engraftment experiments, where a significant reduction in viremia was observed in the WT→*Tlr3*^*−/−*^ chimeras, even though the WT→*Tlr3*^*−/−*^ chimeras still displayed a similar level of joint inflammation comparable to the *Tlr3*^*−/−*^→*Tlr3*^*−/−*^ chimeras (Fig5D–F).

### Impaired virus clearance in *Tlr3*^*−/−*^ mice is due to diminished recognition of CHIKV E2 glycoprotein

**Figure 6:**
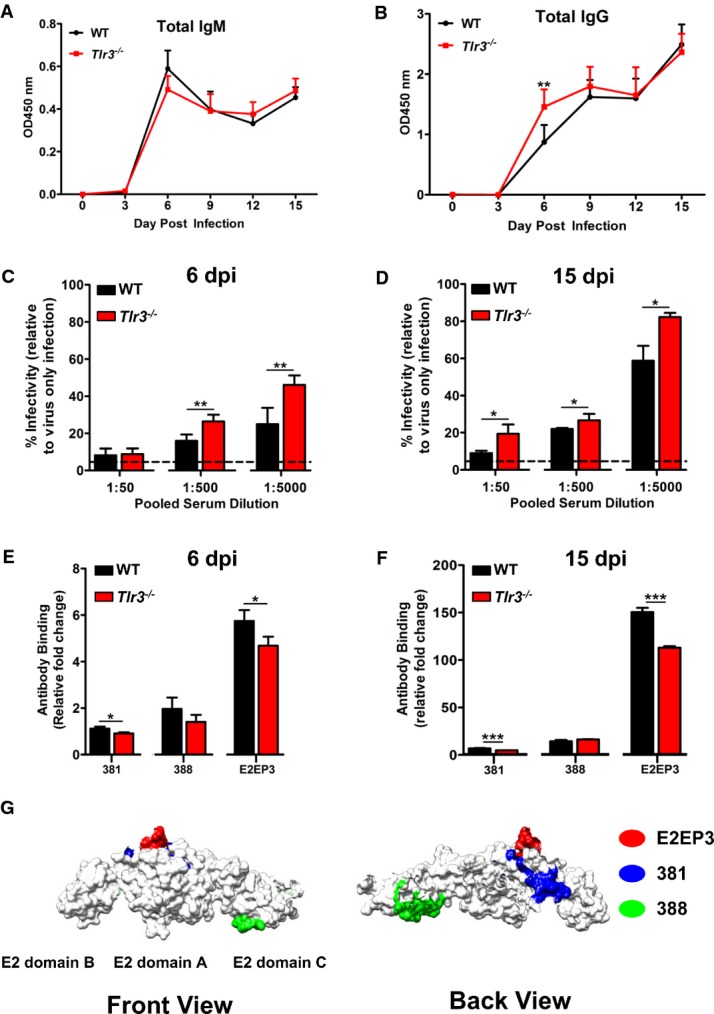
TLR3 modulates early CHIKV-specific IgG response in mice WT and *Tlr3*^*−/−*^ mice (*n *=* *6 per group) were infected with CHIKV (10^6^ PFU) by joint footpad inoculation.A, B Total CHIKV-specific IgM (A) and IgG (B) levels were determined from serum samples collected at 0, 3, 6, 9, 12 and 15 dpi at a dilution of 1:100 and 1:2,000, respectively, using purified CHIKV virion-based ELISA. Data are representative of two independent experiments and presented as mean ± SD (two-tailed Mann–Whitney *U*-test, **P =* *0.0087 6 dpi IgG).C, D Neutralizing capacity of pooled sera collected at 6 dpi (C) and 15 dpi (D) from WT mice is significantly higher than from *Tlr3*^*−/−*^ mice. Pooled sera were diluted 1:50–1:5,000 and mixed with CHIKV (MOI 10) for 2 h before infection of HEK 293T cells for 6 h. Assays were performed in quintuplicate, and data are expressed relative to virus-only-infected samples without sera. Dotted line indicates the detection limit of assay determined from mock-infected samples. Data are representative of two independent experiments and presented as mean ± SD (two-tailed Mann–Whitney *U*-test, **P =* *0.0079 6 dpi 1:500 serum dilution, **P =* *0.0079 6 dpi 1:5,000 serum dilution, *P =* *0.0286 15 dpi 1:50 serum dilution, *P =* *0.0286 15 dpi 1:500 serum dilution, *P =* *0.0286 15 dpi 1:5,000 serum dilution).E, F Mapping antibody reactivity to linear B-cell epitopes within CHIKV E2 proteome. CHIKV E2 epitopes recognized at 6 dpi (E) and 15 dpi (F) were determined in pooled sera collected from infected mice using ELISA specific for overlapping 18-mer linear peptides spanning the CHIKV E2 proteome. The peptide numbers correspond to the position of the 18-mer linear peptides along the CHIKV E2 proteome. Structural data were retrieved from PDB (id: 3N44 and 2XFB) and visualized using the software CHIMERA (Pettersen *et al*, 2004). Assays were performed in triplicate and expressed as relative fold change after normalizing to OD_450_ from non-infected sera. Data are representative of two independent experiments and presented as mean ± SD (two-tailed unpaired *t*-test, *P =* *0.0191 6 dpi epitope 381, *P =* *0.038 6 dpi epitope E2EP3, ***P =* *0.0004 15 dpi epitope 381, ***P =* *0.0001 15 dpi epitope E2EP3).G Localization of identified CHIKV B-cell epitopes (381; blue, 388; green, E2EP3; red) within CHIKV proteome. Epitopes in the E2 glycoprotein were located based on the structural data obtained from PDB records: 3N42. Number corresponds to the region of amino acid sequences in our overlapping 18-mer linear peptides library, along the CHIKV viral genome. All representations are shown in frontal and back view.

Chimera and T-cell depletion experiments suggested a role for TLR3 signaling on B cells in virus control. The role of B cells in mediating virus clearance was demonstrated with CHIKV-specific antibodies that map to epitopes within the CHIKV E1 and E2 glycoproteins (Kam *et al*, 2012b; Lum *et al*, 2013). These antibodies were demonstrated to exhibit high neutralizing activities in both macaque and murine models (Kam *et al*, 2012b, 2014; Lum *et al*, 2013). This led us to further examine whether the impaired virus clearance observed in *Tlr3*^*−/−*^ mice (Fig2A and Supplementary Fig S3C and D) was due to a defective CHIKV-specific antibody response. The levels of CHIKV-specific total IgM and IgG antibodies in sera of infected mice collected at 0, 3, 6, 9, 12 and 15 dpi were first determined using virion-based ELISA (Fig6A and B). CHIKV-specific IgM antibodies were detected on 3 dpi and peaked at 6 dpi with no difference between the WT and *Tlr3*^*−/−*^ mice (Fig6A). CHIKV-specific IgGs were detected later, from 6 dpi, and their levels continued to increase substantially up to 15 dpi (Fig6B). These results indicated that the delayed virus clearance observed in *Tlr3*^*−/−*^ mice was not due to the inability to produce CHIKV-specific antibodies. Interestingly, the level of CHIKV-specific total IgG antibodies was significantly higher in *Tlr3*^*−/−*^ mice than from WT mice at 6 dpi (Fig6B), but this did not translate to a higher CHIKV-neutralizing capacity as demonstrated by the *in vitro* neutralizing assay (Kam *et al*, 2012b) (Fig6C). Intriguingly, serially diluted pooled sera taken at 6 dpi from *Tlr3*^*−/−*^ mice significantly showed *in vitro* lower neutralizing activity against CHIKV compared to sera from WT mice (Fig6C). Similar observations were obtained using pooled sera taken during complete virus clearance at 15 dpi from *Tlr3*^*−/−*^ mice (Fig6D) and *Tlr3*^*−/−*^→*Tlr3*^*−/−*^chimeras (Supplementary Fig S8A). Neutralizing activity was significantly reduced against CHIKV compared to sera from WT and WT→*Tlr3*^*−/−*^ chimeras, respectively.

Studies using plasma samples from CHIKV-infected patients and sera from CHIKV-infected WT mice have revealed that most of the linear B-cell epitopes recognized by CHIKV-specific antibodies are localized in the E2 glycoprotein (Kam *et al*, 2012a,b; Lum *et al*, 2013). Based on these observations, linear B-cell epitopes were screened to assess whether the decreased neutralizing capacity of anti-CHIKV antibodies from *Tlr3*^*−/−*^ mice was associated with diminished epitope recognition. Using the optimized peptide-based ELISA described (Kam *et al*, 2012b; Lum *et al*, 2013), linear peptides covering the E2 glycoprotein proteome (Supplementary Table S1) were screened with diluted sera (1:500) from mice collected at 6 and 15 dpi (Fig6E and F and Supplementary Fig S8B). Linear peptides were screened individually and only three peptides were detected to exhibit significant differences toward the sera between WT and *Tlr3*^*−/−*^ mice (Fig6E and F). Recognition of these three peptides (381, 388 and E2EP3) by CHIKV-specific antibodies from *Tlr3*^*−/−*^ mice was significantly diminished compared to WT mice (Fig6E–G). Specifically, recognition of an early detection serological epitope ‘E2EP3’, which is a dominant linear B-cell epitope in CHIKV-infected patients and animal models (Kam *et al*, 2012a,b; Lum *et al*, 2013), was significantly diminished (FigE–G). Moreover, sera from *Tlr3*^*−/−*^→*Tlr3*^*−/−*^ chimeras were also less neutralizing and similarly associated with a reduced recognition of the same dominant linear E2 epitopes analyzed in *Tlr3*^*−/−*^ mice (Supplementary Fig S8B). These observations strongly indicate that the impaired virus clearance in *Tlr3*^*−/−*^ mice is related to the diminished recognition of dominant linear epitopes in the E2 glycoprotein that are functionally important for virus neutralization (Kam *et al*, 2012b).

### *TLR3* SNP rs6552950 is associated with disease severity and CHIKV-specific IgG neutralizing antibody capacity in CHIKV-infected patients

**Figure 7:**
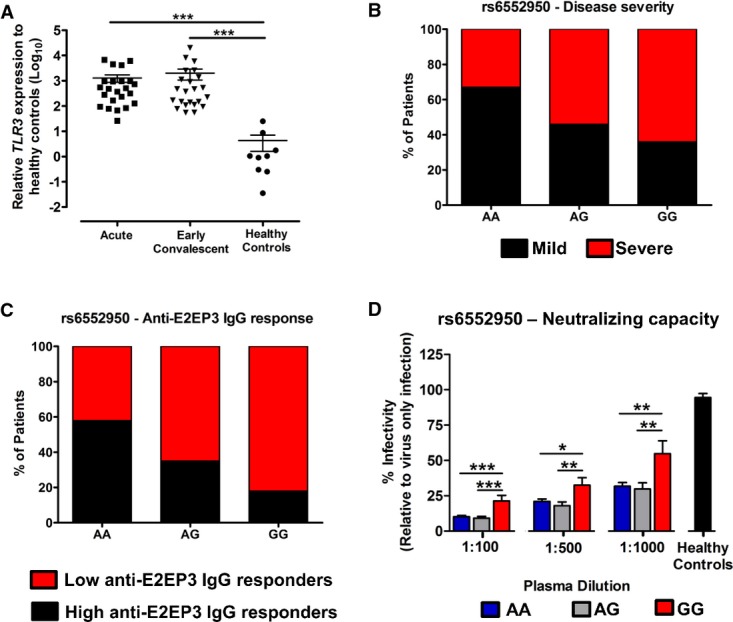
*TLR3* is highly induced in CHIKV-infected patients during the early disease phase and *TLR3* SNP rs6552950 is associated with disease severity and specific IgG response Transcriptional profiles of *TLR3* in PBMCs of CHIKV-infected patients (*n = *23) during acute (median 4 days post-illness onset), and early convalescent (median 10 days post-illness onset) disease. The level of *TLR3* gene expression was expressed relative to healthy controls (*n = *8) after normalization to *GAPDH*. Data are presented as mean ± SEM (two-tailed Mann–Whitney *U*-test, ***P =* *0.0001 acute versus healthy controls, ***P =* *0.0001 early convalescent versus healthy controls).*TLR3* SNP rs6552950 is associated with disease severity in 94 CHIKV-infected patients. Histogram shows the percentage of patients in each genotype exhibiting either mild or severe disease phenotype. Severe illness is defined as patients having either a maximum temperature greater than 38.5°C, a maximum pulse rate greater than 100 beats/min or a nadir platelet count less than 100 × 10^9^/l. Mild illness is defined as patients who do not fulfill these criteria. Association between SNP genotype (AA, *n *=* *43; AG, *n *=* *24; GG, *n *=* *11; unknown, *n *=* *16) and disease phenotype (Mild—AA, *n *=* *29; AG, *n *=* *11; GG, *n *=* *4; Severe—AA, *n *=* *14; AG, *n *=* *13; GG, *n *=* *7) is performed using logistic regression analysis (Table1); *P =* *0.02.*TLR3* SNP rs6552950 is associated with the degree of anti-E2EP3 IgG response in 69 CHIKV-infected patients. Plasma collected at early convalescent (median 10 days post-illness onset) was subjected to E2EP3 peptide-based ELISA at a dilution of 1:2,000. Histogram shows the percentage of patients in each genotype exhibiting either low or high anti-E2EP3 IgG response. Low and high anti-E2EP3 IgG response were defined as being below or above the mean value of anti-E2EP3 IgG response, respectively. Association between SNP genotypes (AA, *n *=* *38; AG, *n *=* *20; GG, *n *=* *11) and anti-E2EP3 IgG response (low—AA, *n *=* *16; AG, *n *=* *13; GG, *n *=* *9; high—AA, *n *=* *22; AG, *n *=* *7; GG, *n *=* *2) is performed using logistic regression analysis; *P =* *0.0376.*TLR3* SNP rs6552950 is associated with the CHIKV-specific antibody neutralizing capacity in 69 CHIKF patients. Plasma collected at early convalescent (median 10 days post-illness onset) was diluted 1:100–1:1,000 and mixed with CHIKV (MOI 10) for 2 h before infection of HEK 293T cells for 6 h. Assays were performed in quadruplicate, and data are expressed relative to virus-only-infected samples without sera. Data are presented as mean ± SEM. Comparison among SNP genotypes (AA, *n *=* *38; AG, *n *=* *20; GG, *n *=* *11) is performed using one-way ANOVA analysis followed by Tukey's multiple comparison test. ***P =* *0.0001 1:100 serum dilution GG versus AA, ***P =* *0.0001 1:100 serum dilution GG versus AG, *P =* *0.0178 1:500 serum dilution GG versus AA, **P =* *0.0056 1:500 serum dilution GG versus AG, **P =* *0.0037 1:1,000 serum dilution GG versus AA, **P =* *0.0041 1:1,000 serum dilution GG versus AG.

To examine the clinical relevance of TLR3-mediated immunity in CHIKV pathogenesis, transcriptional analysis was carried out in PBMCs of patients with CHIKV. *TLR3* levels were significantly higher in CHIKV-infected patients when compared to healthy controls during the acute and early convalescent phases of CHIKV infection (Fig7A). These results suggest that TLR3-mediated signaling forms part of the early innate immune response against CHIKV.

SNP genotyping analysis of 10 *TLR3* tagSNPs was performed in 94 CHIKF patients to establish the impact of TLR3 polymorphisms on CHIKV-induced disease outcome. Association with CHIKV phenotypes revealed that SNP rs3775292 and SNP rs6552950 had significant nominal association to disease occurrence when compared to population controls from the 1000 genome project (Table1) (1000 Genomes Project Consortium *et al*, 2010). In both SNPs, the minor alleles were associated with increased disease susceptibility as indicated by the odds ratios (OR) (Table1). Stratified analysis of these *TLR3* tagSNPs to disease severity revealed that a non-coding variant SNP rs6552950 had a nominal association with disease severity with an OR of 2.39 (*P *<* *0.05) in severe CHIKF patients when compared to mild CHIKF patients (Table1 and Fig7B). In addition, anti-E2EP3 IgG response determined from 69 available patients' plasma in this study cohort further revealed that patients with SNP rs6552950 genotype associated with severe disease outcome and with low anti-E2EP3 IgG response during early convalescence phase (logistic regression analysis; *P *<* *0.05) (Fig7C). Complementing the observations in *Tlr3*^*−/−*^ mice (Fig6A), CHIKV-specific antibody neutralizing assays performed from the plasma of these 69 patients revealed that patients with GG genotype had significantly less neutralizing antibodies as compared to patients with AA or AG genotypes (Fig7D). Taken together, these results suggest that TLR3 polymorphism in humans could influence the neutralizing capacity of CHIKV-specific IgGs by modulating the recognition of CHIKV E2EP3 epitope important for virus neutralization. Specifically, SNP rs3775292 and SNP rs6552950 may be susceptibility factors for CHIKF, with SNP rs6552950 having a possible role in disease severity due to low anti-E2EP3 IgG response.

**Table 1:** Association of *TLR3* SNPs to CHIKV phenotype and severity.

Information of *TLR3* SNPs				Comparison of SNP allele frequency to population controls^a^			Association of SNP with CHIKV disease severity^b^		
SNP^c^	Chromosome^d^	Position^e^	Minor Allele^f^	STAT^g^	*P*-value^h^	OR (L95–U95)^i^	STAT	*P*-value	OR (L95–U95)
rs3775292	4	187003025	C	3.06	0.002*	2.16 (1.31–3.42)	−0.63	0.53	0.80 (0.4–1.6)
rs6552950	4	186994856	G	2.11	0.03*	1.54 (1.03–2.29)	2.39	0.02*	2.31 (1.16–4.57)
rs7657186	4	186994039	A	1.62	0.11	1.43 (0.93–2.21)	−1.32	0.19	0.61 (0.29–1.28)
rs11721827	4	186991137	C	−1.22	0.22	0.70 (0.39–1.24)	−0.20	0.84	0.90 (0.33–2.46)
rs5743312	4	187000256	T	1.08	0.28	1.28 (0.82–2.00)	−0.91	0.36	0.71 (0.33–1.5)
rs7668666	4	187001292	A	0.92	0.36	1.20 (0.81–1.78)	0.12	0.91	1.04 (0.54–2.01)
rs3775291	4	187004074	T	0.82	0.41	1.18 (0.79–1.76)	0.47	0.64	1.15 (0.64–2.09)
rs13108688#	4	186994832	A	−0.42	0.68	0.92 (0.60–1.39)	−0.83	0.41	0.72 (0.33–1.56)
rs3775296	4	186997767	A	0.34	0.73	1.08 (0.71–1.63)	−0.89	0.37	0.74 (0.38–1.45)

a Population controls consist of 60 CEU, 60 CHBJPT and 59 YRI individuals sequenced by the 1000 Genomes Pilot Project.

b ‘Severe disease’ is defined as patients who had either a maximum temperature greater than 38.5°C, a maximum pulse rate greater than 100 beats/min or a nadir platelet count less than 100 × 10^9^/l. ‘Mild disease’ is referred to patients who do not fulfill these criteria.

c SNP, Single nucleotide polymorphism from *TLR3* gene; SNPs rs5743316 and rs5743310 were excluded from further analysis due to failed Sequenom assay design and being monomorphic in control populations, respectively. ^#^SNP rs13108688 was not in Hardy–Weinberg equilibrium in the population.

d,e Chromosome, Position, Chromosome and corresponding position (in base pair) where the SNP is located.

f Minor Allele, The rare allelic form of the SNP variant.

g STAT, Statistic obtained for the regression analysis for the SNP.

h *P*,*P*-value obtained for the regression analysis; ^*^Significant *P*-value < 0.05 is indicated in bold.

i OR(L95–U95), Odds ratio for the SNP as estimated from the minor allele with 95% confidence interval limits.

## Discussion

The detection of PAMPs by PRRs to elicit an inflammatory response is an essential process of the host innate immune response against pathogens (Janeway & Medzhitov, 2002). TLR3-mediated immunity to natural infection in humans has been demonstrated to both limit and exacerbate viral disease progression. It has been shown that defects in TLR3 signaling axis rendered both humans and mice permissive to HSV-1 encephalitis infection (Zhang *et al*, 2007; Pérez de Diego *et al*, 2010; Reinert *et al*, 2012). The immunological control of HSV in the central nervous system was proposed to be mediated through astrocytes which sense HSV-2 in a TLR3-dependent manner and restricts virus replication by inducing anti-viral IFNβ response (Reinert *et al*, 2012). In the case of West Nile virus infection, TLR3 was either protective or deleterious to the host by limiting virus replication in neurons or promoting efficient virus entry into the brain, respectively, depending on the route of inoculation (Wang *et al*, 2004; Daffis *et al*, 2008).

Although studies have emerged demonstrating the functions of RIG-I/MDA5-mediated signaling in anti-CHIKV host response (Schilte *et al*, 2010; White *et al*, 2011; Rudd *et al*, 2012; Olagnier *et al*, 2014), the precise roles of TLRs as PRRs for CHIKV remain unknown. In this study, TLR3 was demonstrated to be a critical PRR in the control of CHIKV replication, immunity and pathology in humans and mice. The increased susceptibility of both *TRIF*^*−/−*^ and *Tlr3*^*−/−*^ primary fibroblasts to CHIKV infection demonstrates a role of TLR3 in controlling CHIKV replication. Furthermore, the loss of TLR3 expression in CHIKV-infected mice significantly increased viremia and exacerbated CHIKV-induced inflammation. However, these findings are in contrast to an earlier report where TLR3 was reported to play a modest role in controlling CHIKV infection in young mice (Schilte *et al*, 2010). This disparity could be due to the age of the animals and the different inoculation routes used. When inoculated intradermally, CHIKV triggered a strong local type I IFN response that was sufficient to locally control virus replication in WT mice and hence no effect on TLR3 signaling (Schilte *et al*, 2010). However, in this study, the effect of TLR3 signaling is apparent because the type I IFN response is independent of TLR3 and therefore insufficient to locally control CHIKV replication during the early phase of the infection.

By tracking virus dissemination using a recombinant FLuc-CHIKV, the loss of TLR3 clearly resulted in exacerbated CHIKV-induced pathology in mice. Moreover, a marked tropism was observed for skeletal muscle, joints and skin in WT and *Tlr3*^*−/−*^ mice. The increased pathology in *Tlr3*^*−/−*^ mice was mediated by an increased infiltration of Ly6G^+^ neutrophils into the infected joint footpad with F4/80^+^ macrophages, which were previously shown to be the primary cellular infiltrates (Gardner *et al*, 2010; Rudd *et al*, 2012; Teng *et al*, 2012). While the exact role of neutrophils in the development of CHIKV-induced inflammation remains unclear, neutrophils were recently implicated in the control of CHIKV infection in mouse (Dhanwani *et al*, 2014; Poo *et al*, 2014) and zebrafish (Palha *et al*, 2013) models. The increased infiltration of myeloid cells is accompanied by the reduction in CD4^+^ T-cell infiltration. However, depletion of CD4^+^ T cells prevented the pathology and abrogated neutrophils without interfering with the development of the viremia. This demonstrated that CD4^+^ T cells are essential for the pathology in WT and *Tlr3*^*−/−*^ mice. One plausible explanation for the exacerbation of joint footpad inflammation in *Tlr3*^*−/−*^ mice is that the loss of TLR3 may reduce the number of anti-inflammatory CD4^+^ T regulatory cells present in the infiltrate. However, this is highly unlikely as demonstrated by bone marrow reconstitution experiments. If TLR3 negatively controlled the number of CD4^+^ T regulatory cells, the WT mice reconstituted with *Tlr3*^*−/−*^ bone marrow would have less CD4^+^ T regulatory T cells. This was not case since WT mice reconstituted with *Tlr3*^*−/−*^ bone marrow had the same level of joint inflammation as the WT mice reconstituted with WT bone marrow.

The dissemination of CHIKV into the brains of *Tlr3*^*−/−*^ mice is of particular interest because, unlike other organs, the brains of WT mice were not infected. CHIKV is not commonly neurotropic, but the occurrence of CHIKV-associated neurological complications has been increasingly reported in patients since the 2006 Indian Ocean outbreak (Rampal *et al*, 2007; Wielanek *et al*, 2007; Das *et al*, 2010; Kashyap *et al*, 2010). Although previous studies in mice have shown that CHIKV dissemination into the central nervous system is dependent on type I IFN signaling (Couderc *et al*, 2008; Abraham *et al*, 2013), it remains to be elucidated whether this is a bystander effect due to overwhelming CHIKV replication or whether the blood–brain barrier has been breached due to the absence of TLR3, permitting virus entry into the brain.

Infection in bone marrow chimeric mice provided evidence on how TLR3-expressing hematopoietic cells are required to elicit an anti-CHIKV innate immune response and regulate CHIKV infection and pathology. Since the role of CD4^+^ T cells was shown to be important for the pathology in the WT and *Tlr3*^*−/−*^ mice via the control of neutrophils recruitment, this suggested that B-cell responses could be defective in *Tlr3*^*−/−*^ mice. Growing evidence has also shown that activation of TLRs promotes B-cell proliferation and IgG isotype class switch (Ruprecht & Lanzavecchia, 2006; Xu *et al*, 2008; Sariol *et al*, 2011). Here, the production of CHIKV-specific IgM and IgG antibodies was not disrupted by the loss of TLR3, and the IgG response was even higher at 6 dpi. Rather, the reduced CHIKV-neutralizing capacity was due to diminished recognition for neutralizing linear B-cell epitopes located in the CHIKV E2 glycoprotein (Kam *et al*, 2012a,b; Lum *et al*, 2013). The loss of TLR3 expression could modulate CHIKV antigen (Ag) processing and presentation by APCs, particularly the immunodominant ‘E2EP3’ epitope, to result in an immunodominance shift (Siddiqui & Basta, 2011). Other virus infection models have demonstrated that TLR3 stimulation in dendritic cells decreases production of nitric oxide which in turn increases proteasomal activity and consequently increases viral antigen processing and presentation (Schwarz *et al*, 2000; Siddiqui *et al*, 2011).

Transcriptional analysis of PBMCs isolated from CHIKV-infected patients revealed that TLR3 expression was up-regulated during the acute and early convalescent phase of the disease. Transcriptional analysis of other PRRs such as RIG-I, MDA5 and IPS-1 (adaptor molecule involved in RIG-I and MDA5 mediated signaling) has shown that these molecules were similarly significantly up-regulated and associated with viral load during acute CHIKV infection (Teng *et al*, 2012), suggesting that the enhanced TLR3 expression is part of a general innate immunity against CHIKV. Previous genetic epidemiological studies have implicated inborn errors in TLR3 immunity and polymorphism in the pathogenesis of HSV-1 encephalitis (Zhang *et al*, 2007; Herman *et al*, 2012; Lafaille *et al*, 2012) and Influenza virus infection (Esposito *et al*, 2012). While the number of CHIKF patients in our study is too small to demonstrate any direct functional link between TLR3 gene polymorphisms and CHIKV disease outcomes, our findings suggest a possible relationship between the presence of *TLR3* SNPs, rs3775292 and rs6552950 with an increased risk of CHIKV disease occurrence. In particular, the SNP rs6552950 polymorphism was demonstrated to be associated with severe CHIKV disease outcome in patients from the Singapore 2008 CHIKV cohort. Antibody response studies in these patients have demonstrated that naturally acquired neutralizing IgG response is dominated by anti-E2EP3 IgG antibodies (Kam *et al*, 2012b). Furthermore, the low anti-E2EP3 IgG response in the SNP rs6552950 GG genotype patients was demonstrated to have reduced neutralizing antibodies, which in turn influenced the severity of disease outcomes in these patients. SNP rs3775292 was previously reported to associate with persisting IgG concentration and serum bactericidal antibody following serogroup C meningococcal polysaccharide–protein conjugate vaccination and an increased risk of colon cancer development (Moore *et al*, 2011; Slattery *et al*, 2011). It will be insightful to assess whether these SNPs would lead to a loss of TLR3 function in larger patient cohorts, including the emergent CHIKV outbreaks in the Caribbean islands (Leparc-Goffart *et al*, 2014).

**Figure 8:**
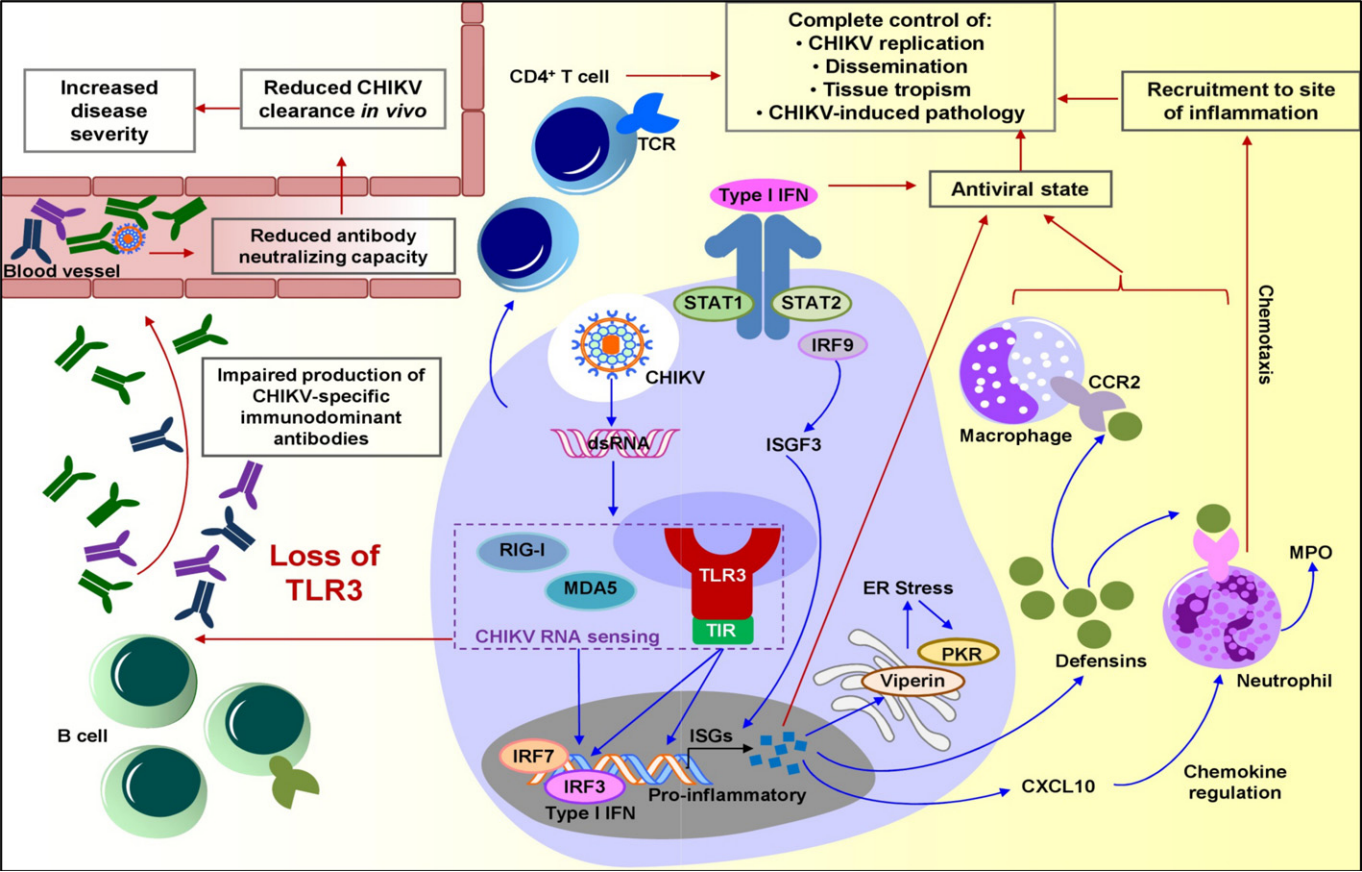
Proposed model for TLR3-mediated anti-CHIKV response during infection Upon CHIKV infection of the target cell, TLR3 functions (blue arrows) in synergy with multiple host cytosolic RNA sensors such as RIG-I and MDA5, to vanguard the coordinated endosomal and cytoplasmic recognition of CHIKV RNA, triggering anti-viral type I IFN response and expression of pro-inflammatory genes. This concerted activation acts to restrict CHIKV replication (red arrows). In parallel, the stimulation of cells will up-regulate TLR3 expression and enhance the anti-CHIKV response. Consequently, activation of TLR3 signaling mediates protection from CHIKV-induced pathology by preventing rapid CHIKV dissemination and restricting tissue tropism (red arrows) tied in with assistance from chemokine regulation (blue arrows). TLR3 signaling can also modulate CHIKV immune recognition during the adaptive immune response to promote effective CHIKV clearance through the production of CHIKV-specific immunodominant antibodies by B cells. Therefore, a loss of TLR3 would lead to a reduction in CHIKV antibody neutralizing capacity due to an impaired production of such antibodies, resulting in the persistence of CHIKV viremia *in vivo* and enhanced/increased disease severity.

Collectively, we provided clear evidences for TLR3 in the control of CHIKV infection. The synergy of TLR3 with multiple host RNA sensors such as RIG-I and MDA5 is indispensable for specific interactions with viral dsRNA to restrict CHIKV replication by inducing a rapid anti-viral type I IFN response together with pro-inflammatory gene expression (Fig8). Essentially, TLR3-mediated immunity controls CHIKV-induced pathology by preventing rapid virus dissemination. The observations reported here also provide further insights on how TLR3-mediated innate responses against CHIKV infection can influence the adaptive immune response, as well as the mechanisms by which TLR3 modulates *in vivo* CHIKV immune recognition. These findings provide a better understanding on one of the key components that induce protective anti-CHIKV responses and will have critical implications in the future development of novel therapeutic strategies against CHIKV and other clinically important alphaviruses that have serious global health impacts.

## Materials and Methods

### Study approval

Blood samples from CHIKF patients used in this study were collected by Institute of Infectious Disease and Epidemiology at Tan Tock Seng Hospital from 1 August through September 23, 2008 with approval from the National Healthcare Group's domain-specific ethics review board (DSRB Reference no. B/08/026). Written informed consent was obtained from all participants in accordance with the Declaration of Helsinki principles. All animal protocols were approved by the Institutional Animal Care and Use Committee (IACUC no. 120714) at the Biological Resource Center at Biopolis, Singapore. All studies involving animals are reported in accordance with the ARRIVE guidelines for reporting experiments involving animals (Kilkenny *et al*, 2010).

### Study population

A total of 94 PCR-confirmed CHIKV-positive individuals were included in this study. There were 48 Chinese, 26 Malay, 10 Indians and 10 of other ethnicity. The median age was 38 with a range of 21 to 67 years of age with 77.7% male and 22.3% female. Based on the clinical parameters defined in Chow *et al* (2011) and Ng *et al* (2009), these individuals were further classified into 53 mild and 41 severe cases. Illness was defined as ‘severe’, if a patient had either a maximum temperature greater than 38.5°C, a maximum pulse rate greater than 100 beats/min or a nadir platelet count less than 100 × 10^9^/l. Patients who do not fulfill these criteria are classified as ‘mild’ (Ng *et al*, 2009; Chow *et al*, 2011).

### Cell culture

African green monkey kidney epithelial cells (Vero-E6), HEK293T, and a mouse hepatocyte cell line (Hepa 1–6) were cultured in DMEM supplemented with 10% FBS (Gibco). Mouse tail fibroblasts were cultured in DMEM supplemented with 10% FBS and 1% penicillin–streptomycin. *Aedes albopictus* mosquito cell line (C6/36) was cultured in Leibovitz's L-15 medium (Life Technologies) supplemented with 10% FBS. All cells were maintained at 37°C with 5% CO_2_, except for the C6/36 cell line which was maintained at 28°C without CO_2_ supplementation.

### Genotyping and association analysis

A total of 11 tagSNPs were identified to cover the linkage disequilibrium block for *TLR3* gene. Genotyping was performed using matrix-assisted laser desorption/ionization time of flight mass spectrometry (MALDI-TOF MS) for the determination of allele-specific primer extension products using Sequenom's MassARRAY system and iPLEX technology (Sequenom Inc). One SNP (rs5743316) failed Sequenom assay design. The design of oligonucleotides was carried out according to the guidelines of Sequenom and performed using MassARRAY Assay Design software. Multiplex PCR amplification of amplicons containing the SNPs of interest was performed using QIAGEN HotStart Taq Polymerase using 5 ng of genomic DNA. Primer extension reactions were carried out according to manufacturer's instructions for iPLEX chemistry. Assay data were analyzed using the Sequenom TYPER software. Clustering of genotype calls was evaluated to determine that the clustering was sufficient for inclusion in the statistical analysis. All SNPs were tested for Hardy–Weinberg equilibrium for quality control and subjected to further statistical analysis. Population controls used for association analysis were the 179 samples sequenced by the 1000 Genomes Pilot Project (60 CEU, 60 CHB+JPT, 59 YRI) (1000 Genomes Project Consortium *et al*, 2010). Sequenom genotypes for SNPs in reverse strand notation (SNPs rs3775291 and rs3775292) were converted to a forward strand notation using the option –flip provided by the software PLINK v1.07 (Purcell *et al*, 2007). One of the 10 *TLR3* SNPs (rs5743310) was monomorphic in two of the three control populations (CEU and CHBJPT) and was therefore excluded from further analysis. Association between the other 9 *TLR3* SNPs and the CHIKV infection phenotype was computed using a logistic regression model. Association between *TLR3* SNPs and severity (mild/severe) was computed using a logistic regression model that included gender and age as covariates. One SNP rs13108688 was not in Hardy–Weinberg equilibrium (*P *<* *0.005) in the population; however, this SNP was not significant for association. Both association analyses were performed using the software PLINK v1.07 (Purcell *et al*, 2007). *P*-values lower than 0.05 were considered significant. The distribution of each *TLR3* SNP genotype and disease severity for each SNP genotype is listed in Supplementary Table S2.

### Virus stocks

CHIKV-IMT isolate used for *in vitro* infections of human cells was isolated from Reunion Island during the 2006 CHIKF outbreak (Bessaud *et al*, 2006). Virus stocks were prepared in Vero-E6 cultures, washed and pre-cleared by centrifugation before storing at −80°C. CHIKV-SGP011 isolate used for *in vitro* and *in vivo* infections in mouse studies was isolated from an outbreak in Singapore in 2008 at the National University Hospital (Her *et al*, 2010) and propagated in Hepa 1–6 and C6/36 cultures, respectively. CHIKV variants expressing FLuc was constructed using a full-length infectious cDNA clone of CHIKV LR2006-OPY1 isolate as described (Tsetsarkin *et al*, 2006; Pohjala *et al*, 2011; Teng *et al*, 2012). Infectious viruses were propagated in C6/36 cultures, washed and pre-cleared by ultracentrifugation before storing at −80°C. Virus titer of all virus stocks used was determined using standard plaque assays with Vero-E6 cells (Her *et al*, 2010; Kam *et al*, 2012b).

### Virus infections

CHIKV infections on primary fibroblasts and continuous cell lines from mouse and human were performed at multiplicity of infection (MOI) 10. Each infection mix consisted of virus suspension prepared in serum-free medium. Viruses were incubated at 37°C and allowed to adsorb for 1.5 h with intermittent shaking before virus inoculum was removed and replaced with complete medium. Cells were incubated at 37°C until harvest at different hpi. Mock infections (medium only) were performed in parallel as controls.

### Flow cytometry and antibodies

Detection of CHIKV Ag was carried out in a two-step indirect intracellular labeling process (Her *et al*, 2010; Teng *et al*, 2012). Data were acquired in BD FACSCanto™ II (BD Bioscience) using BD FACSDiva™ software. Dead cells and duplets were excluded in all analysis with FSC/SSC gating. Results were analyzed with FlowJo version 7.5 software (Tree Star, Inc). Antibodies against CHIKV Ag were purchased from Santa Cruz Biotechnology. Antibodies against mouse CD45 (cat# 557659) and CD8 (cat# 553035) were purchased from BD Bioscience. Antibodies against mouse CD11b (cat# 12-0112-82) was purchased from eBioscience. Antibodies against mouse CD3 (cat# 100200), CD4 (cat# 100531) and Ly6G (cat# 127612) were purchased from Biolegend. Mouse CD4-depleting antibody (cat# BE0003-1) and rat IgG2b isotype control (cat# BE0090) were purchased from Bio X cell.

### Viral RNA extraction and viral load analysis

Viral RNA was extracted using QIAamp® Viral RNA Mini Kit (QIAGEN) according to manufacturer's instructions. Quantification of CHIKV non-structural protein (nsP) 1 negative-sense RNA was determined according to a quantitative real-time PCR (qRT-PCR) TaqMan assay adapted from Plaskon *et al* (Plaskon *et al*, 2009) using QuantiTect® Probe RT-PCR Kit (QIAGEN) in 12.5 μl reaction vol. All reactions were performed using 7900HT Fast Real-Time PCR System machine (Applied Biosciences) with thermal cycling conditions as described previously (Teng *et al*, 2012). The limit of detection was 10 RNA copies/μl.

### Total RNA extraction and gene expression analysis

Total RNA was extracted using RNeasy® Mini Kit (QIAGEN) according to manufacturer's instructions. Quantification of total RNA was performed using NanoDrop 1000 Spectrophotometer (Thermo Scientific), and RNA samples were further diluted to 10 ng/μl. qRT-PCR was performed using QuantiFast™ SYBR® Green RT-PCR Kit (QIAGEN) according to manufacturer's recommendations in 12.5 μl reaction vol. All reactions were performed using 7900HT Fast Real-Time PCR System machine (Applied Biosciences) with thermal cycling conditions as described (Teng *et al*, 2012). The fold change for each gene between CHIKV-infected and mock-infected was calculated as 2^−ΔΔ*C*t^ (Teng *et al*, 2012). The primer sequences of the mouse genes analyzed are listed in Supplementary Table S3.

### Animal studies

Three-week-old WT or *Tlr3*^*−/−*^ C57/BL6 female mice were inoculated s.c. in the ventral side of the right-hind footpad toward the ankle with 10^6^ PFU CHIKV in 25 μl PBS in a non-randomized and non-blinded fashion. Viral RNA extraction was performed from 10 μl of blood collected from the tail, and viremia was determined by qRT-PCR as described (Teng *et al*, 2012; Lum *et al*, 2013; Teo *et al*, 2013). For joint (footpad) inoculated mice, joint inflammation by the measurement of the height (thickness) and the breadth of the footpad using a vernier caliper was calculated as [height × breadth]. The degree of inflammation was expressed as relative increase compared to pre-infection (day 0; d 0) with the following formula: [(*x* − d 0)/d 0] where *x* is footpad size measurements for respective dpi as described (Kam *et al*, 2012b). For joint footpad extraction, mice were sacrificed by terminal anesthesia with ketamine [150 mg/kg]/xylazin [10 mg/kg] followed by intra-cardial perfusion with PBS. Joint footpads were removed and preserved in Trizol (Invitrogen) at −80°C. Tissues were homogenized using a rotor–stator homogenizer (Xiril Dispomix) at 500 *g* for 15 s. Homogenized tissues were transferred to clean tubes and mixed with 230 μl of chloroform. Following 2-min incubation, tissue mixtures were centrifuged at 13,523 *g* for 15 min at 4°C. The aqueous phase was collected, and total RNA isolated as described (Teng *et al*, 2012). For bone marrow chimera, 6-week-old recipient mice were irradiated twice with 600Rad (3 h apart) and i.v. injected with 10^6^ donor bone marrow cells. Absolute CD45^+^ leukocyte blood count was determined at week 4–6 post-reconstitution using flow cytometry and compared to non-chimeric mice of same age. To test for successful adoptive bone marrow cell transfer, *Tlr3* expression in peripheral blood leukocytes from reconstituted mice was determined 6 weeks after reconstitution using quantitative real-time PCR analysis before proceeding with infection. Depletion of CD4^+^ T cells was performed as described (Teo *et al*, 2013). Each mouse was i.p. injected with 500 μg of either CD4-depleting antibody or rat IgG2b isotype control on −2, −1 and 4 dpi. Complete CD4^+^ T-cell depletion was assessed before CHIKV inoculation (day 0).

### *In vivo* imaging

Bioluminescence signals were assessed daily from 1 to 8 dpi and subsequently on every alternate day until 20 dpi using an *in vivo* bioluminescence imaging system (IVIS Spectrum, Xenogen) as described (Teo *et al*, 2013). Luciferin solution containing the luciferase substrate, D-luciferin potassium salt (Caliper Life sciences), was prepared by dissolving in PBS at a concentration of 5 mg/ml. Mice were shaved and anesthetized in an oxygen-rich induction chamber with 2% isoflurane. Bioluminescence signals were measured 2 min after s.c. injection of 100 μl of luciferin solution. Whole-body imaging was performed with the animals in a ventral position, while the feet and head were imaged with the animals in a dorsal position. Bioluminescence imaging was acquired with a field of view (FOV) of 21.7 cm for whole body (FOV-D) and 13.1 cm for foot and head (FOV-C). The mice were exposed for an initial 60 s, followed by a 4-min delay before another exposure at 60 s. When luminescence readings were above the upper detection limit of machine, the exposure time was reduced and kept consistent across groups. Bioluminescence signals of the region of interest were quantified using the Living Image 3.0 software (Caliper Life sciences) and expressed as average radiance (p/s/cm^2^/sr). The lowest detection limit is 0 p/s/cm^2^/sr.

### Histology

Mice were terminally anesthetized with ketamine [150 mg/kg]/xylazin [10 mg/kg] and perfused with PBS by intra-cardial injection. Tissues were fixed in 4% paraformaldehyde (PFA), decalcified and embedded in paraffin wax before 5-μm-thick sections were cut and underwent H&E or immunohistochemical (IHC) staining against mouse Ly6G (Biolegend, cat# 127602), CD11B (AbCam, cat# ab75476) and F4/80 (DAKO, cat# K3468) using established protocols (Teng *et al*, 2012).

### Virion-based ELISA

CHIKV-specific antibody titers were assessed by virion-based ELISA (Kam *et al*, 2012c). Polystyrene 96-well microtiter plates (MaxiSorp, Nunc) were coated with purified CHIKV (10^6^ infectious units per well). Wells were blocked with PBS containing 0.05% Tween 20 and 5% non-fat milk (0.05% PBST + 5% milk) for 1.5 h at 37°C. Serum samples were diluted in 0.05% PBST + 2.5% milk before adding to wells and incubating for 1 h at 37°C. Separately, HRP-conjugated goat anti-mouse IgM (Santa Crus, cat# sc-2064) or IgG (Santa Cruz, cat# sc-2005) were used to detect mouse antibodies bound to virus-coated wells. Reactions were developed using 3,3′,5,5′-tetramethylbenzidine substrate (Sigma-Aldrich) and terminated by Stop reagent (Sigma-Aldrich). Absorbance at 450 nm was measured using a TECAN Infinite® M200 microplate reader and analyzed using Magellan™ software.

### Sero-neutralization

Neutralizing capacity of antibodies from CHIKV-infected mice was analyzed by immunofluorescence-based cell infection assays using HEK 293T cells (Kam *et al*, 2012b). Different amounts of infectious virus required to achieve an infection of MOI 10 were mixed with diluted (1:50–1:5,000), heat-inactivated human plasma or pooled mouse sera and incubated for 2 h at 37°C with gentle agitation (350 rpm). Virus–antibody mixtures were then added to HEK 293T cells seeded on fibronectin-coated 96-well plates and incubated for 1.5 h at 37°C. Virus inoculum was removed and replaced with DMEM supplemented with 10% FBS and incubated for 6 h at 37°C before being fixed with 4% PFA and permeabilized in PBS containing 0.2% Tween 20. Cells were incubated with mouse anti-alphavirus mAb (Santa Cruz, cat# sc-58088) at 1:500 dilution in PBS followed by incubation with Alexa Fluor 488-conjugated goat anti-mouse IgG F(ab’)2 ab (Invitrogen, cat# A11017) at 1:500 dilution in PBS. Nuclei were stained with DAPI (1 μg/μl stock) at 1:10,000 dilution in PBS followed by immunofluorescence quantification using the Cellomics ArrayScan high content analysis reader (Thermo Scientific).

### Peptide-based ELISA

Streptavidin-coated polystyrene 96-well microtiter plates (Pierce, Thermo Scientific) were blocked with 0.1% PBST supplemented with 1% w/v sodium caseinate (Sigma-Aldrich) for 1 h at RT before being coated with biotinylated 18-mer overlapping peptides synthesized based on consensus E2 glycoprotein sequence (Kam *et al*, 2012b) for 1 h at RT. Mouse serum diluted 1:500 (mouse) or human plasma diluted 1:2,000 in 0.1% PBST and 0.1% w/v sodium caseinate were added and incubated for 1 h at RT followed by the addition of relevant HRP-conjugated goat secondary IgG (Santa Cruz, cat# sc-2005) to detect bound antibodies. Reactions were developed using 3,3′,5,5′-tetramethylbenzidine substrate (Sigma-Aldrich) and terminated by Stop reagent (Sigma-Aldrich). Absorbance at 450 nm was measured using TECAN Infinite® M200 microplate reader and analyzed using Magellan™ software.

### Statistical analysis

Statistics were performed using Prism 5.01 (GraphPad Software). Pairwise comparison between infected and mock-infected primary fibroblasts from human and mice was performed using two-tailed unpaired *t*-test. Pairwise comparison between WT and *Tlr3*^*−/−*^ mice in animal studies was performed using two-tailed Mann–Whitney *U-*test. *TLR3* SNPs association analysis (disease severity and anti-E2EP3 IgG response) was performed using logistic regression analysis, while CHIKV-neutralizing capacity association was performed using one-way ANOVA analysis followed by Tukey's multiple comparison test. *P*-values less than 0.05 are considered statistically significant.

**The paper explained**

**Problem**

Chikungunya fever has re-emerged as an important human arboviral infection of global significance, but the factors determining host immunity and pathology are still largely unknown. Toll-like receptors (TLRs) are crucial sensors of virus infection mediated through the recognition of viral nucleic acids, although until now, their role in Chikungunya virus (CHIKV) infection has not been clearly established.

**Results**

The susceptibility to CHIKV infection is markedly increased in both human TRIF-deficient and mouse TLR3-deficient fibroblasts with defective TLR3 signaling. The absence of TLR3 expression in *Tlr3*^*−/−*^ mice resulted in higher viremia and more pronounced joint inflammation due to increased pro-inflammatory myeloid cells infiltration. Mechanistically, infection in bone marrow chimeric mice showed that TLR3-expressing hematopoietic cells are required for effective CHIKV clearance and pointed toward a role for B cells. *Tlr3*^*−/−*^ mice's impaired ability to effectively clear CHIKV was due to a shift in anti-virus antibody specificity that led to a reduced recognition of virus-neutralizing B-cell epitopes by anti-CHIKV IgG. The clinical relevance of TLR3 was further investigated in CHIKV-infected patients, where the level of *TLR3* transcripts was increased in PBMCs of patients. Single nucleotide polymorphism (SNP) genotyping analysis on *TLR3* from 94 patients identified SNP rs6552950 as associated with disease severity and as in mice, a reduced antibody response.

**Impact**

This is the first direct evidence on how TLR3-mediated innate responses against CHIKV infection can influence the adaptive immune response, as well as the mechanisms by which TLR3 modulates *in vivo* CHIKV immune recognition. It is timely, relevant and significant to the field.

## Supporting Information

Supplementary Video S4

Review Preocess File
