# Supplementary material for: Loss of TLR3 aggravates CHIKV replication and pathology due to an altered virus-specific neutralizing antibody response
Source: EMBO Mol Med. 2014 Dec 1;7(1):24–41. doi: 10.15252/emmm.201404459 (PMC4309666; doi:10.15252/emmm.201404459)
Supplement: Supplementary file 1 [file emmm0007-0024-sd1.pdf]

Supplementary Information

Loss of TLR3 aggravates CHIKV replication and pathology due to the regulation of virus-specific neutralizing antibody response

Zhisheng Her, Terk-Shin Teng, Jeslin J.L. Tan, Teck-Hui Teo, Yiu-Wing Kam, Fok-Moon Lum, Wendy W.L. Lee, Christelle Gabriel, Rossella Melchiotti, Anand Kumar Andiappan, Valeria Lulla, Aleksei Lulla, Mar Kyaw Win, Angela Chow, Subhra K. Biswas, Yee-Sin Leo, Marc Lecuit, Andres Merits, Laurent Rénia, and Lisa F.P. Ng

Table of contents:

TABLES:

Supplementary Table 1.
Peptide annotation and amino acid sequences of CHIKV E2 linear epitopes

Supplementary Table 2.
Distribution of genotype for *TLR3* SNPs and severity

Supplementary Table 3.
List of primers used for gene expression analysis

FIGURES:

Supplementary Figure 1.
Gene expression level of type I IFNs and associated molecules in infected mouse WT and *Tlr3*$^{-/-}$ (n = 3 per group) fibroblasts at 12 hpi (MOI 10)

Supplementary Figure 2.
Gene expression of type I IFNs in CHIKV-inoculated joint footpad samples from WT and *Tlr3*$^{-/-}$ mice on 2 dpi

Supplementary Figure 3.
TLR3 modulates CHIKV-induced pathology in mice

Supplementary Figure 4.
TLR3 modulates CHIKV dissemination in mice

Supplementary Figure 5.
TLR3 modulates CHIKV dissemination and tissue tropism in mice

Supplementary Figure 6.
Histological analysis of CHIKV-infected joint footpad from WT and *Tlr3*$^{-/-}$ mice at 3 dpi

Supplementary Figure 7.
Flow cytometry gating strategy for phenotyping of CD3+CD4+, CD3+CD8+ T cells and CD3-CD11b+Ly6G+ neutrophils in CHIKV-infected footpad

Supplementary Figure 8.
Induction of CHIKV-specific antibody response is not dependent on TLR3 in mice

TABLES

Supplementary Table 1. Peptide annotation and amino acid sequences of CHIKV E2 linear epitopes.

Peptide Annotation	Amino Acid[a]	Sequence
350	2793 - 2810	SPHRQRRSTKDNFNVYKA
351	2801 - 2818	TKDNFNVYKATRPYLAHC
365	2913 - 2930	GFTDSRKISHSCTHPFHH
366	2921 - 2938	SHSCTHPFHHDPPVIGRE
367	2929 - 2946	HHDPPVIGREKFHSRPQH
377	3009 - 3026	LTTTDKVINNCKVDQCHA
379	3025 - 3042	HAAVTNHKKWQYNSPLVP
380	3033 - 3050	KWQYNSPLVPRNAELGDR
381	3041 - 3058	VPRNAELGDRKGKIHIPF
388	3097 - 3114	RNMGEEPNYQEEWVMHKK
394	3145 - 3162	GTAHGHPHEIILYYYELY
399	3185 - 3202	AGMCMCARRRCITPYELT
400	3193 - 3210	RRCITPYELTPGATVPFL
401	3201 - 3218	LTPGATVPFLLSLICCIR
402	3209 - 3226	FLLSLICCIRTAKAATYQ
E2EP3	2800 - 2809	STKDNFNVYKATRPYLAHC

[a]The numbers correspond to the amino acid positions along the CHIKV viral genome, with the first amino acid from nsP1 annotated as 1.

Supplementary Table 2. Distribution of genotype for *TLR3* SNPs and severity

				**Disease Severity[a]**					
**SNP[b]**	**Genotype (no. of individuals)**			**Mild**			**Severe**		
rs3775291	AA (13)	AG (40)	GG (40)	AA (6)	AG (24)	GG (23)	AA (7)	AG (16)	GG (17)
rs3775292	CC (56)	CG (32)	GG (6)	CC (31)	CG (17)	GG (5)	CC (25)	CG (15)	GG (1)
rs3775296	GG (62)	GT (21)	TT (8)	GG (35)	GT (12)	TT (6)	GG (27)	GT (9)	TT (2)
rs5743312	CC (61)	CT (29)	TT (4)	CC (33)	CT (17)	TT (3)	CC (28)	CT (12)	TT (1)
rs6552950	AA (43)	AG (24)	GG (11)	AA (29)	AG (11)	GG (4)	AA (14)	AG (13)	GG (7)
rs7657186	AA (4)	AG (35)	GG (55)	AA (3)	AG (22)	GG (28)	AA (1)	AG (13)	GG (27)
rs7668666	AA (8)	CA (30)	CC (52)	AA (4)	CA (18)	CC (30)	AA (4)	CA (12)	CC (22)
rs11721827	AA (73)	CA (16)	CC (1)	AA (42)	CA (9)	CC (1)	AA (31)	CA (7)	CC (0)
rs13108688[#]	AA (5)	AT (22)	TT (51)	AA (4)	AT (13)	TT (29)	AA (1)	AT (9)	TT (22)

[a]"Severe disease" is defined as patients who had either a maximum temperature greater than 38.5ºC, or a maximum pulse rate greater than 100 beats/min, or a nadir platelet count less than 100×10^9 per liter. "Mild disease" is referred to patients who do not fulfill these criteria

[b]SNP – Single nucleotide polymorphism from *TLR3* gene; SNPs rs5743316 and rs5743310 were excluded from further analysis due to failed Sequenom assay design and being monomorphic in control populations respectively. [#]SNP rs13108688 was not in Hardy-Weinberg equilibrium in the population.

Supplementary Table 3. List of primers used for gene expression analysis.

GenBank Accession	Gene	Forward Primer Sequence (5'→3')	Reverse Primer Sequence (5'→3')
		Primers used for qRT-PCR analysis of human genes	
NM_001256799	*GAPDH*	CCACATCGCTCAGACACCAT	GGCAACAATATCCACTTTACCAGAGT
NM_003265	*TLR3*	TTGCCTTGTATCTACTTTTGGGG	TCAACACTGTTATGTTTGTGGGT
		Primers used for qRT-PCR analysis of mouse genes	
NM_008084	*Gapdh*	TTGAGGTCAATGAAGGGGTC	TCGTCCCGTAGACAAAATGG
NM_010503	*Ifnα*	TCATTCTGCAATGACCTCCA	CAGGGGCTGTGTTTCTTCTC
NM_010510	*Ifnb*	CCCTATGGAGATGACGGAGA	TCCCACGTCAATCTTTCCTC
NM_016849	*Irf3*	GGGGAGCCTCTTCACTGAAAACCGTGGA	TAACCACCAGCCTAGACGCAGTCGACAGCA
NM_001252600	*Irf7*	GAAGACCCTGATCCTGGTGA	CCAGGTCCATGAGGAAGTGT
NM_001205314	*Stat1*	TGCTACTGTTCCTTCATATGCAGTATTTCT	ATCTCTTGGTCTTTGTTTACAAAATCCATT
NM_009915	*Ccr2*	TACCTCAGTTCATCCACGGC	GCTCACCATCATCGTAGTCATA
NM_019728	*Defb4*	CCGCTTTTCGACAGATTGGC	CATGGAGGAGCAAATTCTGGC
NC_000074	*Defb14*	TCCAGGGGACGCATTCCTA	ACCGCTATTAGAACATCGACCTA
NM_008361	*Il-1b*	GAAATGCCACCTTTTGACAGTG	TGGATGCTCTCATCAGGACAG
NM_009909	*Il-8r*	ATGCCCTCTATTCTGCCAGAT	GTGCTCCGGTTGTATAAGATGAC
NM_021274	*Ip-10*	GGATGGCTGTCCTAGCTCTG	TGAGCTAGGGAGGACAAGGA
NM_009140	*Mip2*	CTCTCAAGGGCGGTCAAAAAGTT	TCAGACAGCGAGGCACATCAGGTA
NM_010824	*Mpo*	AGTTGTGCTGAGCTGTATGGA	CGGCTGCTTGAAGTAAAACAGG
NM_126166	*Tlr3*	CACAGGCTGAGCAGTTTGAA	TTTCGGCTTCTTTTGATGCT

FIGURES

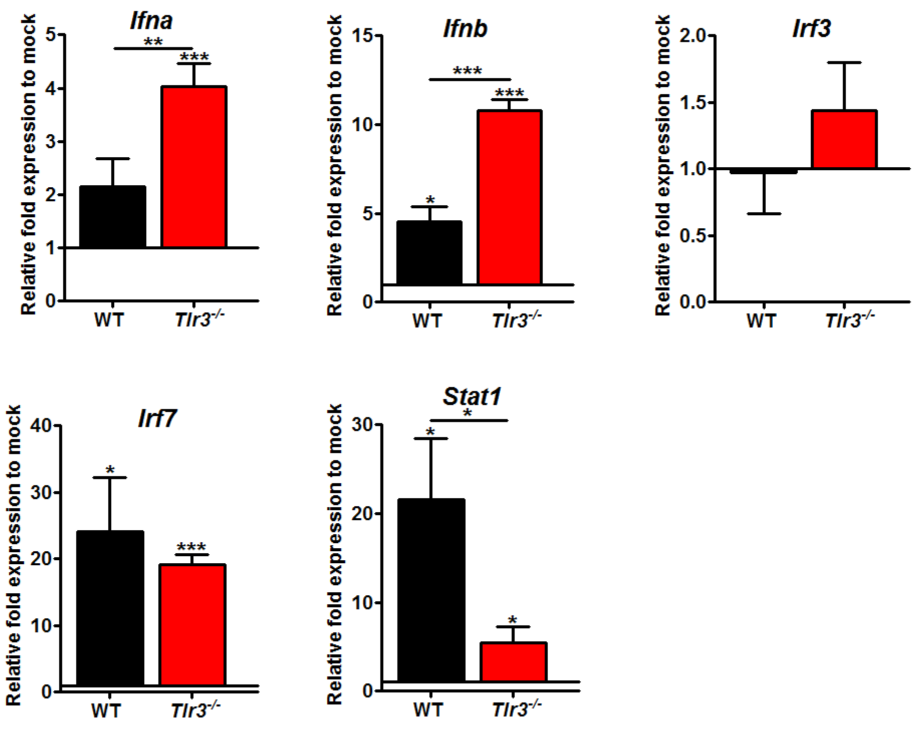

Supplementary Figure 1. Gene expression level of type I IFNs and associated molecules in infected mouse WT and *Tlr3*$^{-/-}$ (*n* = 3 per group) fibroblasts at 12 hpi (MOI 10). The level of gene expression was expressed relative to mock-infected fibroblasts after normalization to *Gapdh*. Infections were performed in triplicate and data are representative of two independent experiments and presented as mean ± SD (Pair-wise comparison to mock-infected fibroblasts was performed using two-tailed unpaired t-test, ****P* = 0.0007 *Ifna Tlr3*$^{-/-}$, **P* = 0.0132 *Ifnb* WT, ****P* = 0.0001 *Ifnb Tlr3*$^{-/-}$, **P* = 0.0323 *Irf7* WT, ****P* = 0.0001 *Irf7 Tlr3*$^{-/-}$, **P* = 0.0279 *Stat1* WT, **P* = 0.0143 *Stat1Tlr3*$^{-/-}$. Pair-wise comparison between infected WT and *Tlr3*$^{-/-}$ fibroblasts was performed using two-tailed unpaired t-test, ***P* = 0.0082 *Ifna*, ****P* = 0.0005 *Ifnb*, **P* = 0.0169 *Stat1*).

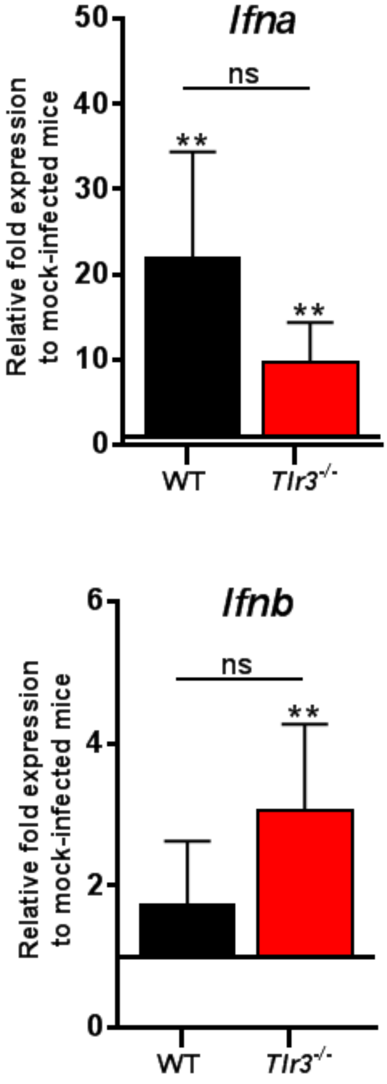

Supplementary Figure 2. Gene expression of type I IFNs in CHIKV-inoculated joint footpad samples from WT and *Tlr3$^{-/-}$* mice on 2 dpi. WT and *Tlr3$^{-/-}$* mice (n = 5 per group) were infected with CHIKV (10^6 PFU) by joint footpad inoculation. The level of gene expression was expressed as fold change compared to mock-infected mice footpad (n = 5 per group) after normalization to *Gapdh*. Data are representative of two independent experiments and are presented as mean ± SD (Pair-wise comparison to mock-infected mice was performed using two-tailed Mann Whitney *U* test, $^{**}P$ = 0.0079 *Ifna* WT, $^{**}P$ = 0.0079 *Ifna Tlr3$^{-/-}$*, $^{**}P$ = 0.0079 *Ifnb Tlr3$^{-/-}$*. Pair-wise comparison between infected WT and *Tlr3$^{-/-}$* mice was performed using two-tailed Mann Whitney *U* test., non-significant for *Ifna* and *Ifnb*).

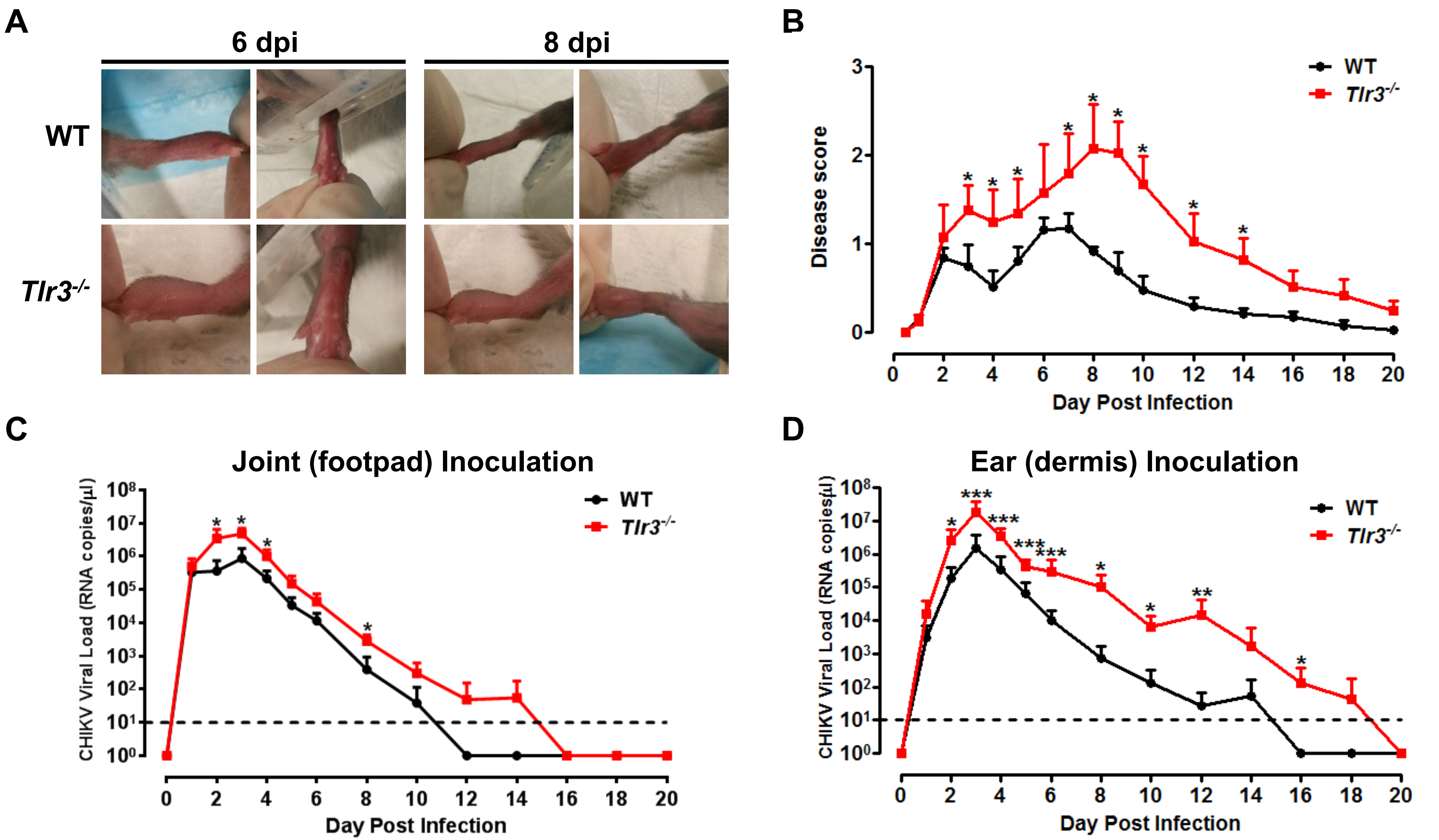

Supplementary Figure 3. TLR3 modulates CHIKV-induced pathology in mice. WT and *Tlr3*$^{-/-}$ mice were infected with FLuc-CHIKV (10^6 PFU) by either joint footpad (*n* = 4-5 per group) (A- C), or ear (dermis) (*n* = 9-10 per group) (D) inoculation. (A) Images of CHIKV-induced joint inflammation at 6 dpi and 8 dpi. (B) Extent of joint inflammation was measured daily and expressed as disease score relative to day 0 (pre-infection). (C,D) Viremia was determined in blood collected from the tail with viral load quantification. Data are representative of two independent experiments and presented as mean ± SD (Two-tailed Mann Whitney *U* test, **P* = 0.0397 3dpi disease score, **P* = 0.0159 4dpi disease score, **P* = 0.0159 5dpi disease score, **P* = 0.0317 7dpi disease score, **P* = 0.0159 8-14dpi disease score, **P* = 0.0159 2dpi viremia footpad, **P* = 0.0317 3dpi viremia footpad, **P* = 0.0159 4dpi viremia footpad, **P* = 0.0317 8dpi viremia footpad, **P* = 0.0433 2dpi viremia ear, ****P* = 0.0006 3dpi viremia ear, ****P* = 0.0002 4dpi viremia ear, ****P* = 0.0002 5dpi viremia ear, ****P* = 0.0001 6dpi viremia ear, **P* = 0.0133 8dpi viremia ear, **P* = 0.0133 10dpi viremia ear, ***P* = 0.0041 12dpi viremia ear, **P* = 0.0158 16dpi viremia ear). Dotted line indicates the limit of viral load detection at 10 CHIKV RNA copies/µl.

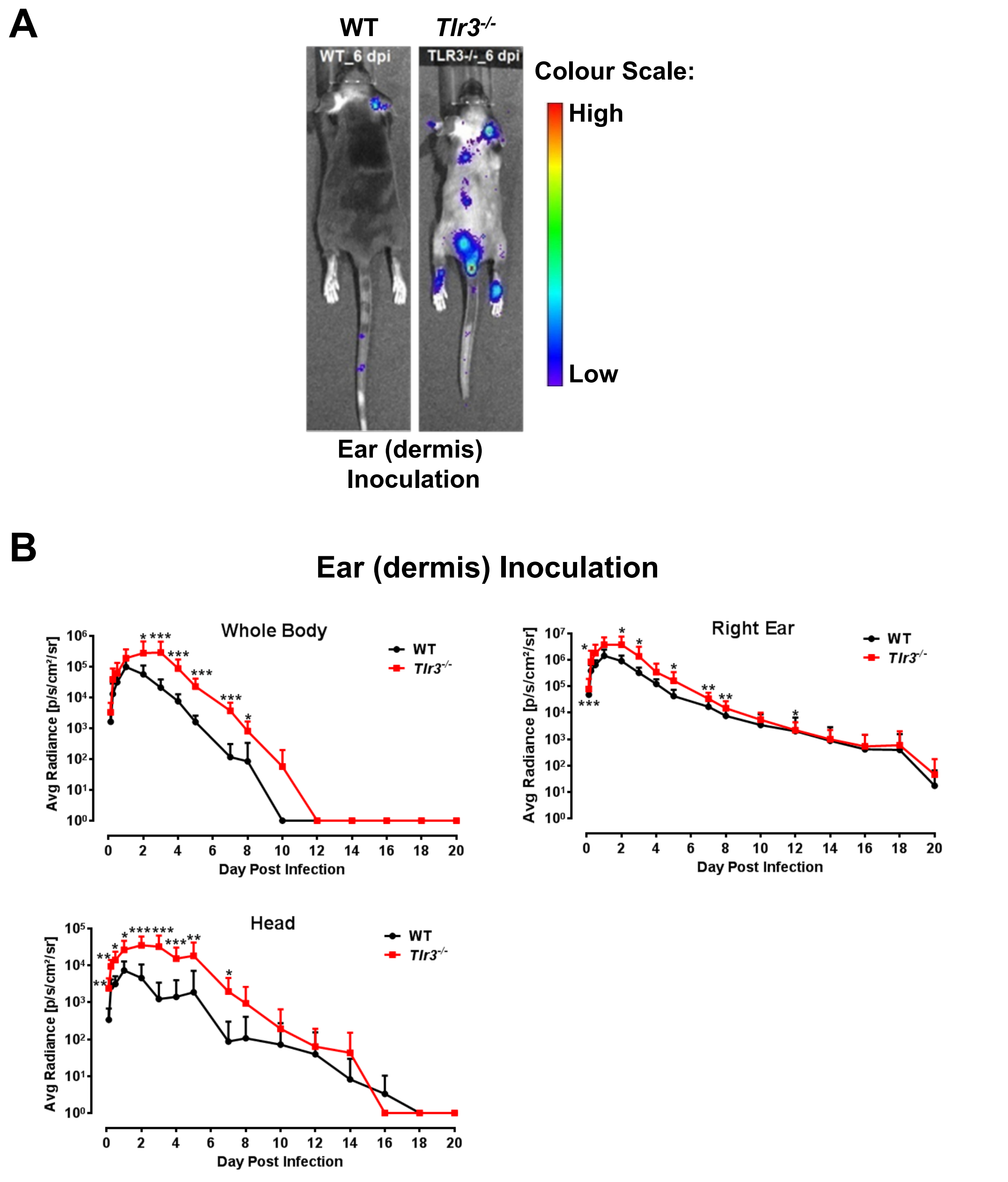

Supplementary Figure 4. TLR3 modulates CHIKV dissemination in mice. WT and *Tlr3*$^{-/-}$ mice were infected with FLuc-CHIKV (10^6 PFU) by ear (dermis) inoculation. Bioluminescence signals were measured using an *in vivo* bioluminescence imaging system. (A) Images of representative 6 dpi WT and *Tlr3*$^{-/-}$ mice (*n* = 9-10 per group) infected by ear (dermis) inoculation. Color scale indicates the level of bioluminescence signals detected. (B) Bioluminescence signals of whole body, head region and at site of inoculation were quantified and expressed as average radiance (p/s/cm^2/sr). The lowest limit of detection is 0 p/s/cm^2/sr. Data are representative of two independent experiments and are presented as mean ± SD (Two-tailed Mann Whitney *U* test, **P* = 0.0279 2 dpi whole body, ****P* = 0.0004 3 dpi whole body, ****P* = 0.0004 4 dpi whole body, ****P* = 0.0002 5 dpi whole body, ****P* = 0.0009 7 dpi whole body, **P* = 0.0144 8 dpi whole body, ****P* = 0.0009 3 hpi right ear, **P* = 0.022 6 hpi right ear, **P* = 0.0279 2 dpi right ear, **P* = 0.0349 3 dpi right ear, **P* = 0.0101 5 dpi right ear, ***P* = 0.0076 7 dpi right ear, ***P* = 0.0074 8 dpi right ear, **P* = 0.0272 12 dpi right ear, ***P* = 0.0021 3 hpi head, ***P* = 0.0015 6 hpi head, **P* = 0.0101 12 hpi head, **P* = 0.0296 1 dpi head, ****P* = 0.0002 2 dpi head, ****P* = 0.0001 3 dpi head, ****P* = 0.001 4 dpi head, ***P* = 0.0012 5 dpi head, **P* = 0.0173 7 dpi head).

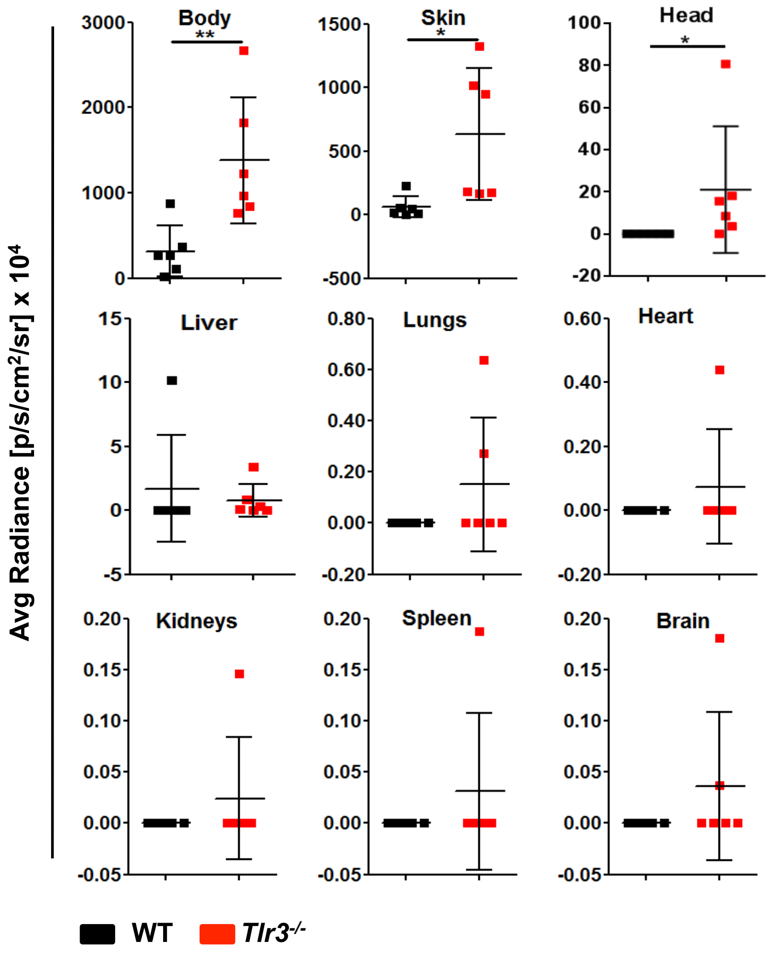

Supplementary Figure 5. TLR3 modulates CHIKV dissemination and tissue tropism in mice. WT and *Tlr3*$^{-/-}$ mice were infected with FLuc-CHIKV (10^6 PFU) by joint footpad (*n* = 6 per group) inoculation. Bioluminescence signals were assessed using an *in vivo* bioluminescence imaging system. Bioluminescence signals of various organs, body and skin of infected WT and *Tlr3*$^{-/-}$ mice at 6 dpi were quantified and expressed as average radiance (p/s/cm^2/sr). The lowest limit of detection is 0 p/s/cm^2/sr. Data are representative of two independent experiments and presented as mean ± SD (Two-tailed Mann Whitney *U* test, ***P* = 0.0087 body, **P* = 0.0152 skin, **P* = 0.0152 head).

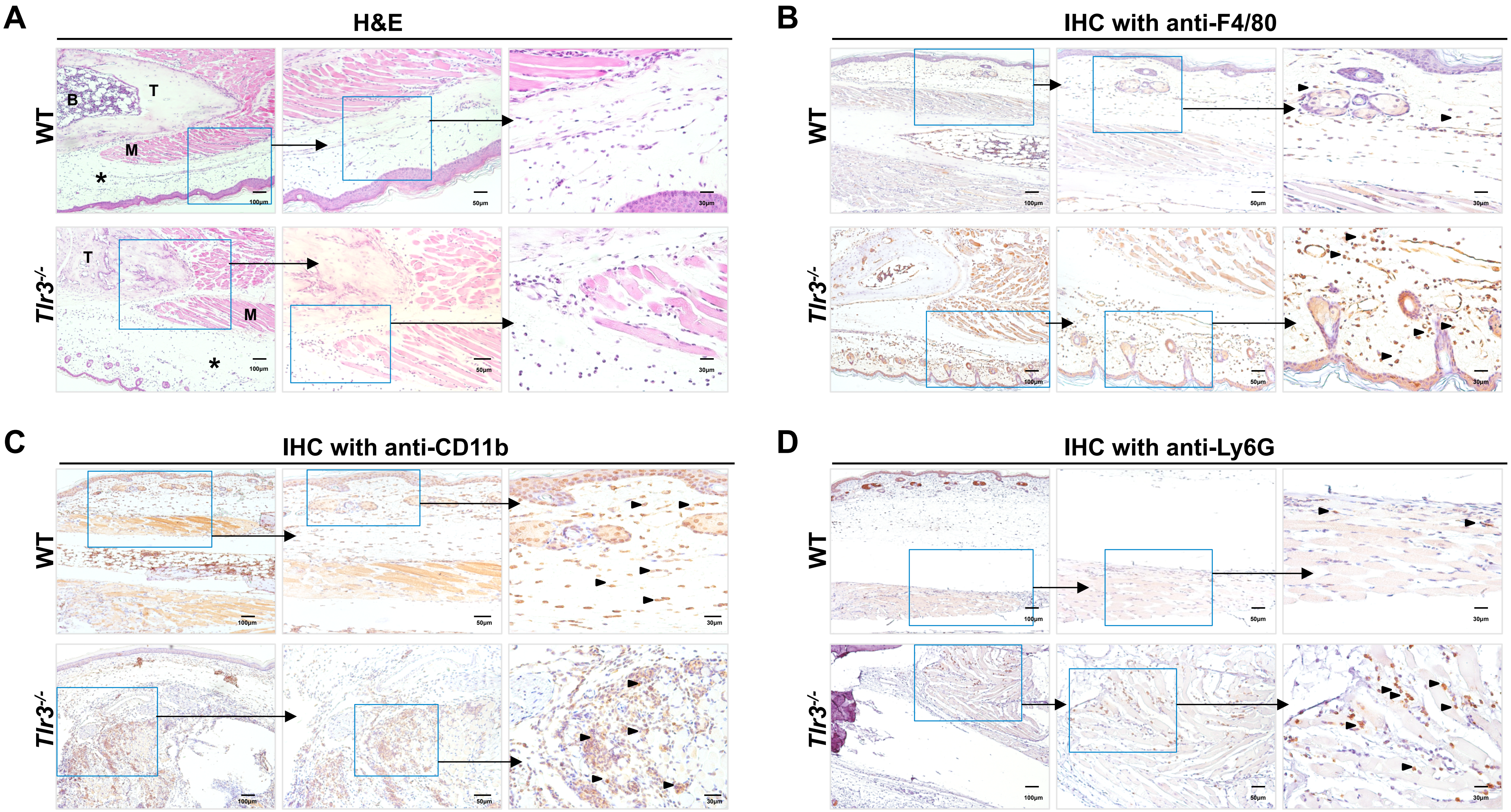

Supplementary Figure 6. Histological analysis of CHIKV-infected joint footpad from WT and *Tlr3*$^{-/-}$ mice at 3 dpi. Sections of CHIKV-infected joint footpad were analyzed by H&E staining (A), and IHC labeling with anti-F4/80 antibody (B), anti-CD11b (C) and anti-Ly6G (D). Boxed regions are shown at higher magnification on the right. * = edema, B = bone, M = muscle, T = tendon. Black arrows indicate positively stained cells. Images presented are representative of 3 mice per group from two independent experiments.

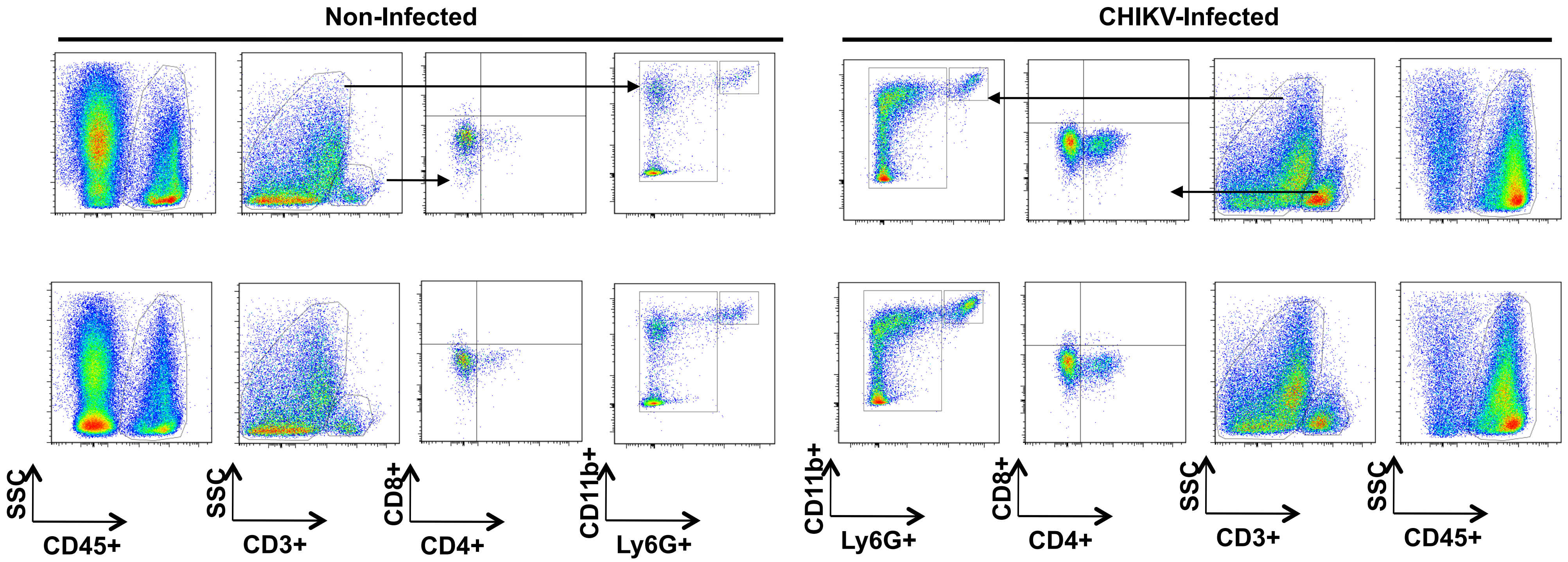

Supplementary Figure 7. Flow cytometry gating strategy for phenotyping of CD3+CD4+, CD3+CD8+ T cells and CD3-CD11b+Ly6G+ neutrophils in CHIKV-infected footpad. WT and *Tlr3*$^{-/-}$ mice were infected with CHIKV (10^6 PFU) by joint footpad (n = 6 per group) inoculation. At 6 dpi, cells from spleen, popliteal lymph node, and footpad were harvested and labeled for CD45, CD3, CD4, CD8, CD11b and Ly6G. CD3+ cells were gated from live CD45+ cells before separated according to CD4+ and CD8+ populations respectively.

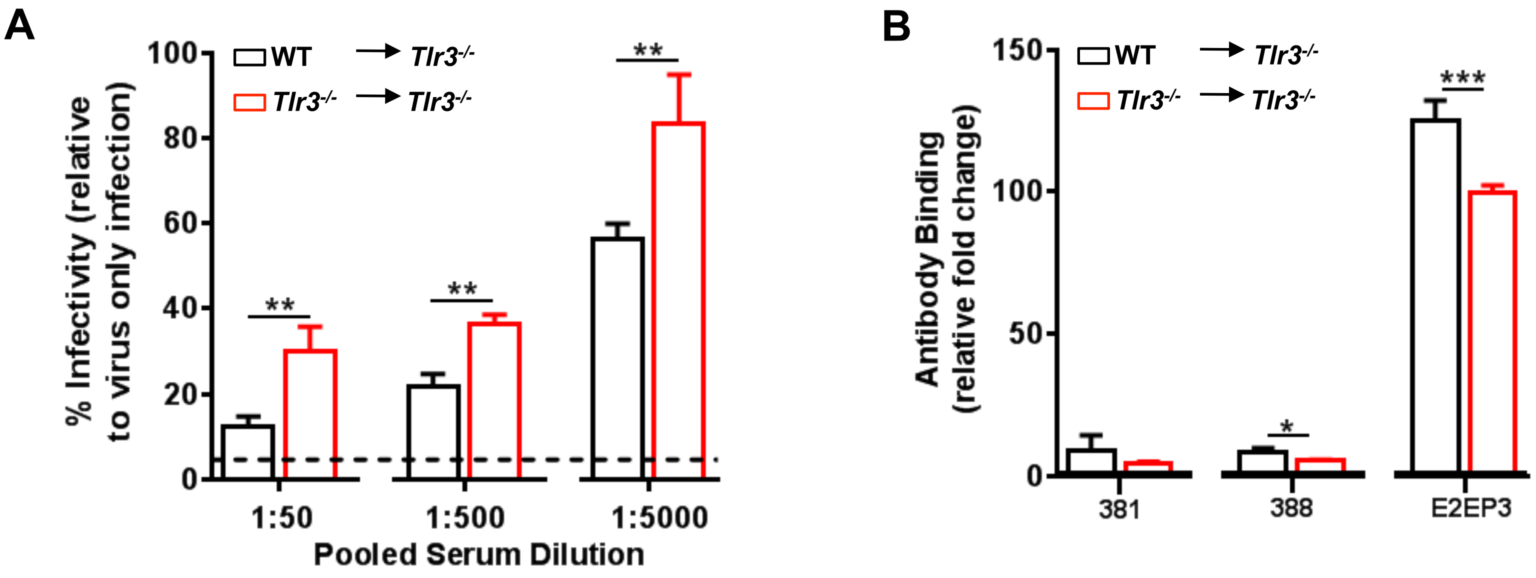

Supplementary Figure 8. Bone marrow engraftment of WT hematopoietic cells into *Tlr3*$^{-/-}$ recipient mice improved neutralizing capacity of pooled sera. WT →*Tlr3*$^{-/-}$ and *Tlr3*$^{-/-}$→*Tlr3*$^{-/-}$ chimeras (*n* = 6 per group) were infected with CHIKV (10^6 PFU) by joint footpad inoculation. (A) Neutralizing capacity of pooled sera collected at 15 dpi from WT →*Tlr3*$^{-/-}$ chimeras is significantly higher than from *Tlr3*$^{-/-}$→*Tlr3*$^{-/-}$ chimeras. Pooled sera were diluted 1:50 - 1:5000 and mixed with CHIKV (MOI 10) for 2 h before infection of HEK 293T cells for 6 h. Assays were performed in quintuplicate and data are expressed relative to virus only-infected samples without sera. Dotted line indicates the detection limit of assay determined from mock-infected samples. Data are representative of two independent experiments and presented as mean ± SD (Two-tailed Mann Whitney *U* test, ***P* = 0.0079 1:50 serum dilution, ***P* = 0.0079 1:500 serum dilution, ***P* = 0.0079 1:5000 serum dilution). (B) Mapping antibody reactivity to linear B-cell epitopes within CHIKV E2 proteome. CHIKV E2 epitopes recognized at 15 dpi were determined in pooled sera collected from infected mice using ELISA specific for overlapping 18-mer linear peptides spanning the CHIKV E2 proteome. The peptide numbers correspond to the position of the 18-mer linear peptides along the CHIKV E2 proteome. Assays were performed in triplicate and expressed as relative fold change after normalizing to OD_{450} from non-infected sera. Data are representative of two independent experiments and presented as mean ± SD (Two-tailed unpaired t-test, **P* = 0.0170 epitope 388, ****P* = 0.0006 epitope E2EP3).
