# Supplementary material for: Loss of TLR3 aggravates CHIKV replication and pathology due to an altered virus-specific neutralizing antibody response
Source: EMBO Mol Med. 2014 Dec 1;7(1):24–41. doi: 10.15252/emmm.201404459 (PMC4309666; doi:10.15252/emmm.201404459)
Supplement: Supplementary file 6 [file emmm0007-0024-sd6.pdf]

EMBO Molecular Medicine

Loss of TLR3 aggravates CHIKV replication and pathology due to an altered virus-specific neutralizing antibody response

Zhisheng Her, Terk-Shin Teng, Jeslin J.L. Tan, Teck-Hui Teo, Yiu-Wing Kam, Fok-Moon Lum, Wendy W.L. Lee, Christelle Gabriel, Rossella Melchiotti, Anand Kumar Andiappan, Valeria Lulla, Aleksei Lulla, Mar Kyaw Win, Angela Chow, Subra K. Biswas, Yee-Sin Leo, Marc Lecuit, Andres Merits, Laurent RÈnia, and Lisa F.P. Ng

Corresponding author: Lisa Ng, Singapore Immunology Network

Review timeline:

Submission date:	22 July 2014
Accepted:	03 November 2014

Editor: Céline Carret

Transaction Report:

No Peer Review Process File is available with this article, as the authors have chosen not to make the review process public in this case.

© EMBO
